# Epidemiology, treatment patterns, and outcomes in gestational trophoblastic neoplasia: a systematic review

**DOI:** 10.3389/fonc.2026.1923113

**Published:** 2026-10-14

**Authors:** Mairead Kearney, Jason Hoffman, Laura Cilek, Jason Simeone

**Affiliations:** 1 Merck Healthcare KGaA, Darmstadt, Germany; 2 EMD Serono Research & Development Institute, Inc., Billerica, MA, United States, an affiliate of Merck KGaA; 3 GIPAM GmbH, Wismar, Germany

**Keywords:** epidemiology, gap analysis, gestational trophoblastic neoplasia, outcomes, systematic literature review, treatment patterns

## Abstract

**Introduction:**

Current global data on epidemiology, treatment patterns, outcomes, and care pathways for patients with gestational trophoblastic neoplasia (GTN) are needed.

**Methods:**

A systematic review included publications from Embase and Medline (interventional: 2005–2025; observational: 2021–2025) and conference abstracts (2022–2024). Benign gestational trophoblastic disease (GTD) studies, case reports, and case series were excluded. Treatment response rates and 95% CIs were calculated from aggregated data.

**Results:**

Of 117 included studies, 66.7% (n=78) were cohort studies; 54.7% (n=64) were conducted in Asia. GTN incidence from GTD varied from 6.2% in the UK to 16.8% across Asian studies. Methotrexate and dactinomycin were the most common real-world first-line treatments in low-risk GTN. Second-line and high-risk patients’ treatment included diverse multiagent regimens and immunotherapy. Across trials, first-line treatment response with dactinomycin (82.9%) was higher than methotrexate (70.5%); in observational studies, response was similar and inversely associated with GTN risk score. Nausea and other gastrointestinal events were more common with dactinomycin than methotrexate.

**Discussion:**

Incidence of GTN varied by region and setting. Consensus supports methotrexate or dactinomycin as first-line therapy in low-risk GTN. Treatment options were more diverse in high-risk GTN and later-line regimens. Unmet needs to improve response rates and minimize toxicity persist.

## Highlights

- An SLR of the most recent publications on GTN found higher rates of GTN in Asia than in other geographic regions
- Single-agent MTX and Act-D were commonly used as first-line treatment for low-risk GTN, but unmet needs still exist
- Treatment recommendations were more heterogeneous for patients with high-risk disease and second-line therapy

## 1 Introduction

Gestational trophoblastic neoplasia (GTN) encompasses a number of rare, malignant tumors, including invasive moles (IM), choriocarcinoma, placental-site trophoblastic tumors (PSTT), and epithelioid trophoblastic tumors (ETT) (1). Approximately 50% of patients with GTN have a prior molar pregnancy as the antecedent event, 25% had a tubal pregnancy or miscarriage, and 25% of GTN cases follow preterm or term pregnancies (2). Among molar pregnancies, persistent GTN develops in about 15% to 20% of complete hydatidiform moles and 0.5% to 5% of partial hydatidiform moles (3, 4).​ The epidemiology of GTN appears to vary based on geographic region, with a higher incidence in Asia than Europe and North America (5).​ Other GTN types, including IM, PSTT, and ETT, are likely much rarer.

Following diagnosis, patients are evaluated to determine disease severity, allowing GTN classification via an International Federation of Gynecology and Obstetrics (FIGO) anatomic stage and World Health Organization (WHO) risk score. These classifications can be used to understand the likelihood of disease progression and resistance to treatment with single-agent chemotherapy ​ (6).​ Patients with FIGO stage I or stage II/III GTN and a WHO prognostic score <7 are classified as having low-risk disease and are treated with single-agent chemotherapy, typically either methotrexate (MTX) or dactinomycin (Act-D); the majority of patients with GTN fall into this category ​ (1)​. Patients with stage IV GTN or a WHO score ≥7 are classified as having high-risk disease and are treated with multiagent chemotherapy, although there is less consensus on the most appropriate regimen for these patients ​ (1).

Initial treatment of low-risk GTN with MTX or Act-D is currently associated with complete normalization of human chorionic gonadotropin (hCG) hormone (i.e., GTN eradication) in approximately 60-90% of patients, indicating a substantial unmet need in remaining patients ​ (7)​. The safety and efficacy of avelumab, an anti–PD-L1 monoclonal antibody, was assessed in the investigator-initiated TROPHAMET phase I/II trial as first-line therapy for low-risk GTN in combination with MTX ​ (8). Among 26 patients assessed for the efficacy endpoint, 25 (96.2%) experienced hCG normalization after a median treatment duration of 3.32 months (9). Other completed and ongoing trials have found clinically meaningful results when evaluating the use of immune checkpoint inhibitors, including avelumab, pembrolizumab, and camrelizumab (alone or in combination with chemotherapy or anti-angiogenic agents) in the treatment of high-risk, chemoresistant, and recurrent GTN (9–12).

In the context of this dynamic treatment landscape, a systematic literature review (SLR) was performed to understand the current global epidemiology, treatment patterns, clinical outcomes, professional guideline recommendations, and unmet needs among patients with GTN.

## 2 Methods

The objectives of this SLR were to summarize: (1) the epidemiology of GTN; (2) current treatment patterns; (3) clinical outcomes, including treatment response, relapse, subsequent pregnancies, overall survival, and adverse events; (4) guideline-recommended treatment regimens; and (5) information related to the patient journey with GTN.

Relevant publications were identified via an extensive literature search across Embase and Medline through Ovid on January 28, 2025. In addition, a supplemental search was performed for key conference presentations from 2022 to 2024 (American Society of Clinical Oncology, European Society of Gynecologic Oncology [ESGO], European Society for Medical Oncology, FIGO, International Society for the Study for Trophoblastic Diseases [ISSTD], and Society of Gynecologic Oncology).

A systematic search strategy was developed to identify relevant publications using the Population, Intervention, Comparator, Outcome, Study Design, and Time (PICOS-T) framework (**Supplementary Table 1**). The study selection process was grounded in the application of the predefined eligibility criteria, ensuring that only studies meeting the prespecified standards were included in the review. In general, English-language articles, focused on patients with GTN, that reported study outcomes of interest and were either interventional (from the last 20 years, to include key trials for drugs used in the management of GTN) or observational (from the last 5 years, to restrict real-world data to the most relevant information for contemporary clinical practice), or consisted of treatment guidelines (i.e., the most recent guidelines from relevant organizations) were selected for inclusion. Articles reporting on studies performed in nonhumans or *in vitro*, articles on benign gestational trophoblastic disease (GTD), case reports, and case series were excluded.

The selection of relevant publications followed a structured, multi-step process. First, the relevance of all identified publications was assessed via title and abstract screening. Publications that did not meet the established eligibility criteria were then excluded. Publications deemed potentially relevant after title and abstract screening proceeded to full-text review. Two independent reviewers conducted each step of the selection process to ensure objectivity. Publications were also screened for duplicate results in the same patient populations. In case of disagreement, a third reviewer (mediator) was consulted to resolve any conflicts.

Relevant patient characteristics were extracted from the literature when available and as reported. Stratified data were extracted as reported (e.g., by age and risk score). Relevant outcomes included GTN incidence, treatment patterns, regimens by treatment line, treatment response, recurrence, overall survival, and outcomes of subsequent pregnancies (successful births or losses). Two researchers independently completed data extraction based on predefined data extraction tables. Any deviations were resolved by a mediator.

Quality and bias assessments were performed using the Newcastle Ottawa Scale ​ (13) for cohort and case-control studies; the National Heart, Lung, and Blood Institute Quality Assessment Tool for cross-sectional studies (14); Risk of Bias in Non-Randomized Studies – of Interventions (13) for nonrandomized interventional studies; the Risk of Bias in Randomized Trials (15) for randomized interventional studies; and the Assessing the Methodological Quality of Systematic Reviews version 2 for SLRs (16).

### 2.1 Statistical methods

Descriptive statistics summarizing the information extracted from reviewed studies were analyzed and reported, including measures such as means and associated standard deviations (SDs), medians, ranges, frequencies, and percentages. Missing data were not imputed; rather, this information was tabulated as part of the gap assessment to identify information not available in current literature.

## 3 Results

A total of 1,899 studies were initially screened for study inclusion through the review of study titles and abstracts; of these, 265 (14.0%) were retained for full-text review (**Figure 1**).

**Figure 1:**
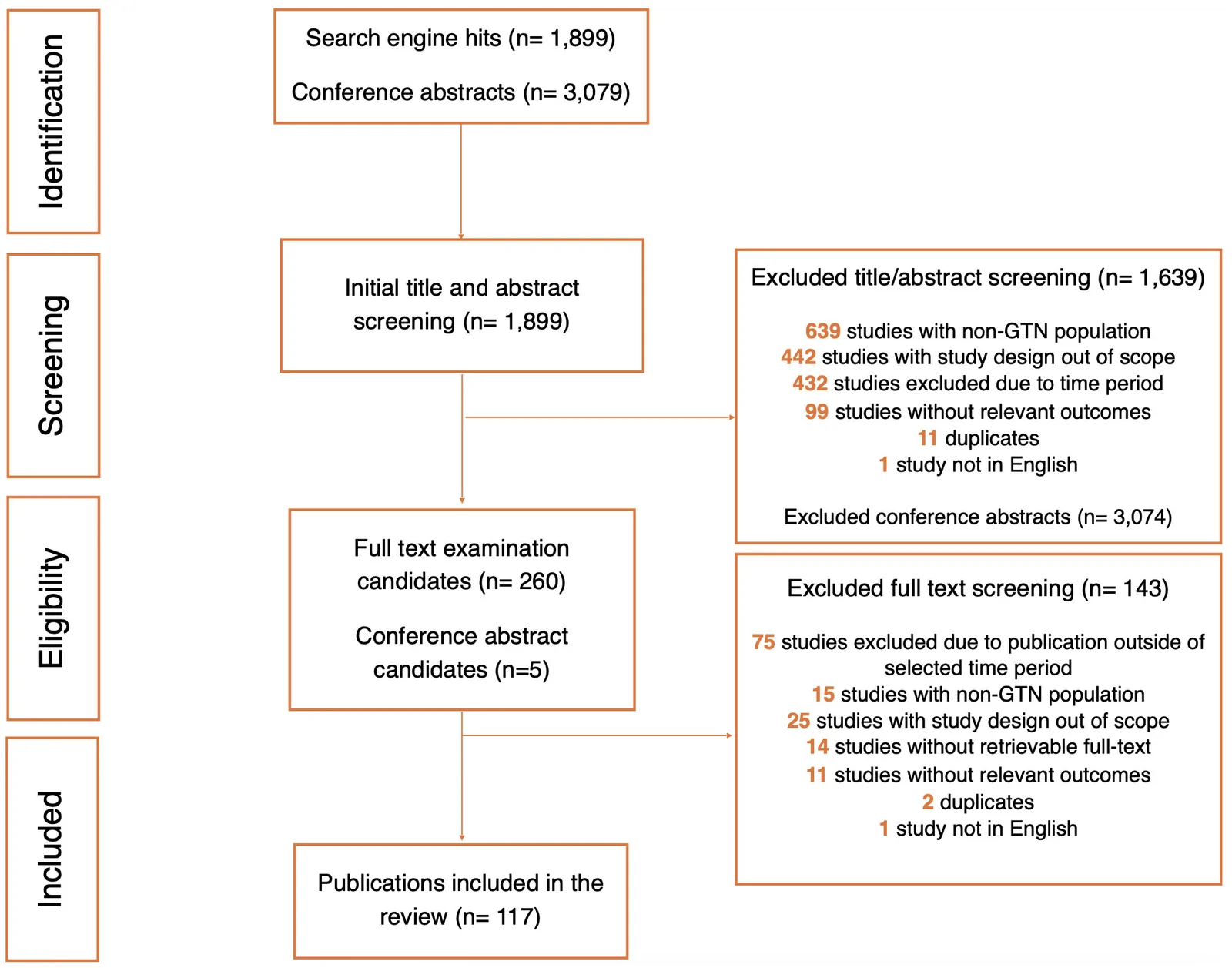
PRISMA diagram of study selection. GTN, gestational trophoblastic neoplasia; PRISMA, Preferred Reporting Items for Systematic Reviews and Meta-Analyses.

Following full-text review, 117 studies (44.2%) met all criteria for inclusion (**Supplementary Table 2**). Of the 148 studies excluded at this stage (full-text studies and conference abstracts), reasons for exclusion were as follows: 75 (50.7%) due to publication dates, 25 (16.9%) due to study design, 15 (10.1%) due to study population, 14 (12.2%) due to unavailability of full-text articles, and 11 (7.4%) due to study outcomes being out of scope; two duplicate studies and one study not in English language were excluded. No conference abstracts met all criteria for study inclusion.

### 3.1 Study characteristics

Of the 117 included studies, cohort studies were most common (n=78; 66.7%). Others included interventional studies (n=14; 12.0%), SLRs (n=8; 6.8%), cross-sectional studies (n=6; 5.1%), hybrid observational studies (n=2; 1.7%), guidelines (n=6; 5.1%), and a case-control study (n=1; 0.9%). Most studies originated from Asia (n=64; 54.7%), followed by Europe (n=14; 12.0%), North America (n=9; 7.7%), multiple countries (n=7; 6.0%), and South America (n=6; 5.1%).

Excluding guidelines and SLRs (n=14), hospitals were the most common study setting, comprising 89.3% (n=92/103) of all observational and interventional studies. Five studies (4.3%) were conducted using national registries, and three (2.6%) were conducted using regional registries. Three studies used other or multiple data sources.

Study sample size varied widely. Among clinical trials, the mean sample size was 60.2 patients (SD: 44.7); the median was 49 patients (range: 4–149). For observational studies, the mean sample size was 17, 410 patients (SD: 155,623), while the median sample size was 113 patients (range: 13–1,418,096). Among SLRs, the mean number of studies included was 26.4 (SD: 19.8); the median was 23 studies (range: 5–70).

The study populations assessed in the included studies also varied; 10 studies (8.5%) focused on patients diagnosed with choriocarcinoma (n=957), 6 (5.1%) focused on patients with ETT (n=175), 7 (6.0%) included patients with PSTT only (n=329), and 29 (24.8%) reported the development of post-molar GTN of any type (i.e., hydatidiform mole (HM), IM, complete and partial mole, HM with coexisting fetus; n=3,057).

### 3.2 Baseline characteristics

Across studies reporting continuous age (n=53), the mean of mean ages was 33.0 years, and means ranged from was 26.75 to 51.3 years (**Supplementary Figure 1**). The mean of median ages was similar (33.2 years).

A total of 31 studies (26.5%) reported the previous pregnancy type for 7,249 patients; however, the manner in which the antecedent pregnancy was identified varied, including specific types of molar pregnancies, ectopic pregnancies, and outcomes including abortions and miscarriages. In six studies reporting information about miscarriage in the antecedent pregnancy, 238 of 1,843 (13.0%) pregnant patients who experienced miscarriage were diagnosed with GTN of varying risk levels and types (17–22).

A total of 47 observational studies reported GTN FIGO staging. Staging was influenced by the selection criteria of studies and therefore is not a generalizable distribution of GTN by stage at diagnosis. Included articles reported on heterogenous study populations, such as patients classified as having high or low risk, or focused more narrowly on special subtypes of GTN such as PSTT or ETT, which are generally classified as high risk. FIGO scoring at baseline from reporting studies is summarized in **Supplementary Figure 2**; note that some studies reported FIGO stage groupings, while others reported single stages.

Forty-five observational studies reported WHO scores, of which 25 reported continuous scores. Six studies reported mean WHO scores (median: 10.1), while 19 studies reported median scores (median: 3). The large difference in risk scores reported across studies is likely due to diverse study selection criteria (e.g., some studies selected only high-risk patients) as well as differences in the way that risk scores were calculated during the study period (23). ​Forty-two studies categorically reported WHO scores, which were regrouped into categories of low risk (0–6) and high risk (7+) when possible. In total, 8,225 patients could be reclassified; 6,346 (77.2%) were classified as low risk, and 1,879 (22.8%) were classified as high risk.

### 3.3 Epidemiology

A total of 34 studies reported GTN incidence, including 20 studies in Asia. GTN incidence was calculated from diverse patient populations, including those within specific risk groups or subtypes of GTN, all pregnant patients, and patients diagnosed with GTD, including specific GTD subtypes.

Four studies reported the incidence of patients with unspecified GTN as a proportion of the population with GTD (n=705/11,149); the percentage of patients with GTN among all those with GTD ranged between 6% and 43% (24–27). One large study conducted in the UK monitored 10,402 patients with GTD between 1998 and 2018, accounting for over 93% of patients with GTD among the four studies; of these, 643 patients were diagnosed with GTN (incidence: 6.2%) (27). Nine studies in Asia reported overall and type-specific GTN incidence from GTD (226 patients with GTN from 1,342 patients with GTD; pooled percentage: 16.8%) (24, 26).

Thirteen studies reported GTN incidence following partial or complete molar pregnancies; some studies reported both measures. The GTN incidence estimates following molar pregnancy ranged from 0.26% in a study from a French GTD reference center between 1999 and 2020 (28) to 48.9% in a study from a teaching hospital in Bangkok between 2007 and 2020 (29).

Fourteen studies, eight of which were conducted in Asia, reported on the development of GTN following undefined molar pregnancies (i.e., partial or complete status not specified), including 877 patients with GTN from a total of 11,946 patients with molar pregnancies (7.3%) (25–38). Among the subset of Asian studies, 327 patients developed GTN out of 1,436 with molar pregnancies (22.8%) (26, 27, 29, 31, 32, 35–37).

From four studies, the pooled incidence of GTN following complete molar pregnancy was 14.5% (1,073/7,410) pregnancies. The highest reported incidence estimate was among women of older maternal age; one study in Vietnam reported a GTN incidence of 34.7% over 3.25 years among women with GTD aged >40 years (36).

Three studies reported the incidence of GTN following partial HM; two of these studies reported the number of patients with GTN as well as the number of women with partial moles (the pooled GTN incidence was 21 occurrences among 2,766 patients with partial molar pregnancies [0.8%]) (25, 28, 36). The highest estimate reported GTN incidence of 29.9% in Vietnamese women with GTD aged >40 years (36).

Four studies reported a pooled choriocarcinoma incidence of 0.003% from 33 of 1,052,475 deliveries (26, 27, 39, 40).

### 3.4 Treatment patterns

A total of 81 studies specified treatment information, including 14 clinical trials and 67 observational studies.

Among the clinical trials, 13 specified first-line treatment information, and three specified second- or later-line information (**Supplementary Table 3**). Nine trials used MTX, and six used Act-D in the first-line setting. One study evaluated a combination of MTX and avelumab in the first-line setting (8), and one trial evaluated a combination of immunotherapy drugs (nivolumab and ipilimumab) among four patients with refractory GTN (41).

Among all observational studies, 49 specified first-line treatment regimens (**Supplementary Table 4**), and 21 studies provided information on second- or later-line treatments. Of studies that reported first-line chemotherapy information, 33 reported use of MTX, 15 reported use of Act-D, and 36 reported unspecified or other regimens.

In observational studies, patients who experienced first-line treatment failure with single-agent chemotherapy were often treated by switching to either Act-D or MTX (from initial MTX or Act-D treatment) or to a multiagent regimen such as etoposide, MTX, Act-D, cyclophosphamide, and vincristine (EMA-CO). One observational study reported that patients were treated with pembrolizumab following resistance with first-line chemotherapy (42).

### 3.5 Treatment response

#### 3.5.1 Treatment response in clinical trials

Outcomes associated with clinical response were reported in 14 clinical trials (**Supplementary Table 5**). Response outcomes included remission, response, hCG normalization, and successful treatment, which may have been presented without complete, partial, or other disease-specific qualifiers. A summary of treatment response outcomes in low-risk patients is presented in **Figure 2**. Considering all risk groups, the percentage of patients who received first-line Act-D regimens in clinical trials and achieved a measure of treatment response (82.9%) appeared higher than the percentage of patients who received first-line MTX regimens in clinical trials (70.5%). Across all trials and all reported agents, the pooled percentage of patients achieving treatment response was 79.4%.

**Figure 2:**
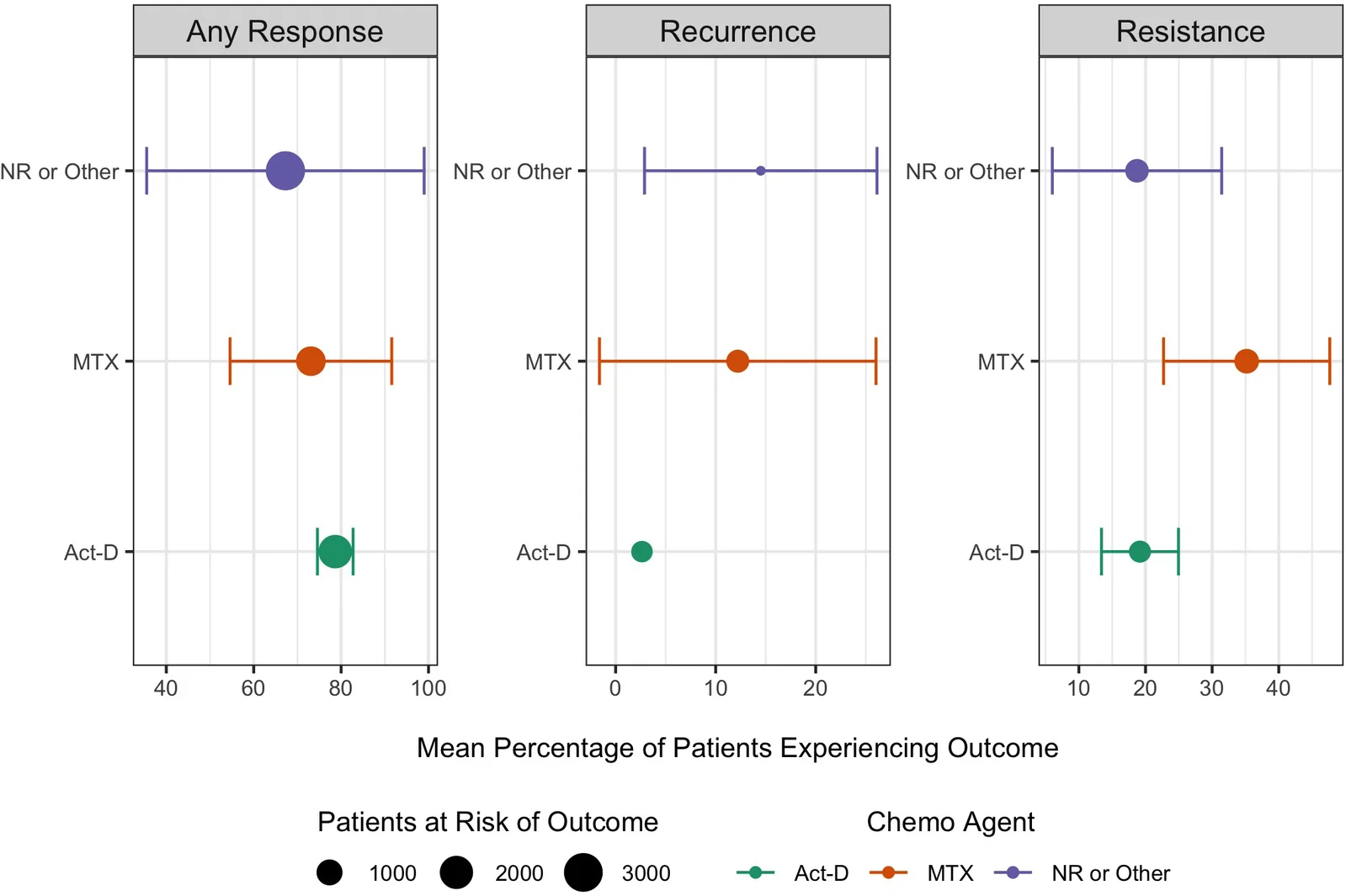
Treatment response among all first-line patients with low-risk GTN in interventional studies. Act-D, dactinomycin; GTN, gestational trophoblastic neoplasia; MTX, methotrexate; NR, not reported.

#### 3.5.2 Treatment response in observational studies

A total of 41 observational studies reported a general treatment response (without specifying complete or partial response). As expected, response to treatment varied by regimen, line of treatment, and GTN risk, among other factors (**Table 1**; **Supplementary Table 6**). Among these studies, 52.9% of patients achieved an unspecified response to treatment across all treatments, all lines, and all GTN risk categories. Treatment response appeared to be associated with GTN risk score. For example, treatment response following first-line Act-D therapy among low-risk patients was 54.0% across four studies, while the treatment response to first-line Act-D among high-risk patients was 38.7% in two studies. Response to second-line therapy among low-risk patients was 92.4% (SD: 15.3%) in three studies, while response to second-line therapy among high-risk patients was 62.8% (SD: 40.9%).

**Table 1:** Summarized clinical responses to first-line treatment reported in observational studies.

Clinical response	Drug	Risk	Studies, n	Patients at risk of given outcome, n	Patients with response, n (%)	Patients with response in studies reporting given outcome, mean (SD), %
Any response	Act-D	Low	4	1,705	920 (53.96)	65.34 (37.3)
	Act-D	High	2	2,001	774 (38.68)	23.61 (36.32)
	Act-D	NR	2	48	33 (68.75)	0
	MTX	NR	6	676	404 (59.76)	67.16 (10.42)
	MTX	Low	6	1,368	1,052 (76.9)	64.68 (36.1)
	Other	Low	6	399	295 (73.93)	74.71 (25.23)
	Other	High	9	603	354 (58.71)	58.79 (34.38)
	Other	NR	10	630	367 (58.25)	54.32 (36.89)
Complete response	Act-D	Low	3	1,026	818 (79.73)	77.71 (4.4)
	Act-D	High	1	994	764 (76.86)	76.86 (NA)
	Act-D	NR	1	18	15 (83.33)	83.33 (NA)
	MTX	Low	3	978	705 (72.09)	79.85 (17.77)
	MTX	NR	2	477	265 (55.56)	62.95 (19.86)
	Other	Low	2	93	78 (83.87)	86.62 (14.65)
	Other	High	3	233	217 (93.13)	88.4 (14.27)
	Other	NR	3	204	182 (89.22)	86.27 (10.55)
Partial response	Other	Low	1	31	9 (29.03)	29.03 (NA)
	Other	NR	1	66	6 (9.09)	9.09 (NA)
Chemoresistance	Act-D	Low	1	570	86 (15.09)	15.09 (NA)
	Act-D	High	1	994	48 (4.83)	4.83 (NA)
	Act-D	NR	1	43	10 (23.26)	23.26 (NA)
	MTX	Low	1	170	71 (41.76)	41.76 (NA)
	MTX	NR	5	684	269 (39.33)	33.86 (13.47)
	Other	Low	2	276	87 (31.52)	37.86 (17.17)
	Other	High	3	288	89 (30.9)	30.03 (17.34)
	Other	NR	5	476	69 (14.5)	13.26 (3.05)
Recurrence	Act-D	Low	1	570	15 (2.63)	2.63 (NA)
	Act-D	High	2	1,007	28 (2.78)	5.67 (7.59)
	MTX	Low	2	190	7 (3.68)	3.56 (3.57)
	MTX	NR	4	507	56 (11.05)	18.69 (15.57)
	Other	High	1	87	6 (6.9)	6 (NA)
	Other	NR	4	92	16 (17.39)	14.5 (11.62)
Mortality	Act-D	Low	1	570	0	0 (NA)
	Act-D	High	2	1,007	10 (0.99)	5.86 (9.37)
	MTX	Low	1	20	2 (10)	0
	Other	High	2	107	11 (10.28)	7.22 (6.74)
	Other	NR	2	106	9 (8.49)	5 (7.07)

Act-D, dactinomycin; MTX, methotrexate; NA, not applicable; NR, not reported; SD, standard deviation.

Seventeen studies reported complete treatment response, while four studies reported partial response (**Table 1**; **Supplementary Table 7**). Across all 17 studies reporting complete response (across all therapy lines, regimens, and risk categories), 77.0% of patients achieved complete response. Across all lines of treatment and GTN risk categories, the mean percentage of patients achieving complete response to Act-D was 78.7% (SD: 4.1%), while the corresponding mean percentage for patients who received MTX was 73.1% (SD: 18.5%).

#### 3.5.3 Chemoresistance in observational studies

A total of 16 observational studies reported data on chemoresistance (**Supplementary Table 8**). Across all lines of treatment, regimens, and GTN risk categories the mean reported chemoresistance rate was 14.4% (SD: 9.2) across three Act-D studies and 35.2% (SD: 12.5) across six MTX studies.

#### 3.5.4 Mortality in observational studies

Ten studies reported mortality after treatment among patients with GTN (**Supplementary Table 9**). In one study of low-risk patients receiving Act-D (n=570), mortality was not observed. In two studies of high-risk patients receiving Act-D (n=1,007), a mean of 5.9% (SD: 9.4%) of patients died during the follow-up period, which varied in length across studies. The mortality rate in patients with low-risk receiving first-line MTX treatment appeared high (10%); however, this measure was only reported in one study that included 20 patients.

#### 3.5.5 Recurrence in observational studies

Eight studies reported GTN recurrence after complete response to therapy (**Supplementary Table 10**). Across all lines of treatment, regimens, and risk categories, 14.4% (SD: 15.1) developed recurrent GTN. The mean percentage of Act-D treatment across lines and risk categories was 4.9% (SD: 6.4%), while the corresponding percentage for MTX was 9.1% (SD: 10.1%). Patients who received therapies other than Act-D or MTX had the highest risk of recurrence across lines and categories of GTN risk (23.5%; SD: 17.2%). Patients with high-risk GTN who were treated with Act-D appeared to have a higher risk of recurrence vs patients with low-risk GTN, with a mean of 5.7% vs 2.6%, respectively.

### 3.6 Subsequent pregnancies in clinical trials and observational studies

Two clinical trials assessing Act-D, EMA-CO, and floxuridine, dactinomycin, etoposide and vincristine (FAEV) reported on subsequent pregnancies (43, 44). Among seven FAEV-treated and three EMA-CO–treated patients who ceased contraception use, eight and three pregnancies were reported in each respective treatment group (43). In a trial assessing pulsed Act-D therapy, six of eight subsequent pregnancies in women receiving second-line Act-D treatment were successful (44).

Ten observational studies and one SLR reported on subsequent reproduction following treatment for GTN. Five studies reported information on the number of pregnancies following treatment (in 156 of 372 patients) (17, 18, 45–47). From five studies, 105 successful live births were reported from 131 pregnancies (80.2%) among women treated for GTN. (18, 33, 45, 46, 48).

### 3.7 Patient journey–related outcomes

A total of 45 studies reported information about patient journey, including seven clinical trials and 38 observational studies. The most frequently reported measures included information on diagnosis (n=25 observational studies; n=3 clinical trials), such as time to diagnosis and/or treatment initiation (**Supplementary Table 11**). Other reported patient journey–related outcomes included subsequent unrelated diseases (n=1); experiences before, during, and/or after chemotherapy (n=2); GTN originally misdiagnosed as other conditions (n=1) (49); and treatment facility type (n=3).

One study in Brazil reported on the positive relationship between a patient’s proximity to a treatment center and the likelihood of an earlier diagnosis of lower stage GTN and successful treatment (50), while two studies, one each in Switzerland and Canada, assessed the importance of GTD reference centers by comparing outcomes of patients initially treated at a GTD reference center to those referred from elsewhere (51, 52).

### 3.8 Summary of recent GTN treatment guidelines

All guidelines included (n=5; **Supplementary Tables 12** and **13**) recommend single-agent chemotherapy (MTX and Act-D when specified) for first-line treatment of low-risk GTN. The most commonly mentioned multiagent chemotherapy regimen was EMA-CO, which is generally recommended for use as first-line treatment in high-risk GTN or following treatment failure in first-line low-risk GTN. Surgery was recommended for high-risk or aggressive forms of GTN (e.g., PSTT and ETT) in conjunction with the patient’s preference for preserving future fertility.

Four of the five guidelines reviewed (from the National Comprehensive Cancer Network; Royal College of Obstetricians and Gynaecologists; Society of Obstetrician and Gynaecologists of Canada; and Gynecologic Societies from Germany, Austria, and Switzerland) acknowledged the emerging use of immunotherapies as an alternative or additional treatment for cancers, including GTN (1, 3, 53, 54). The guidelines generally state that the initial and long-term effectiveness of such immunotherapy drugs in the GTN treatment setting is not evaluated. Guidelines from the Society of Obstetrician and Gynaecologists of Canada also acknowledge the clinical trial evaluating avelumab in combination with MTX (54).

### 3.9 Adverse events

#### 3.9.1 Adverse events from clinical trials

Eleven clinical trials reported adverse events (AEs), of which eight specified the grade of AE (**Figure 3**; **Supplementary Table 14**). Some trials reported specific AEs associated with treatment, while others reported AEs as general categories, such as “gastrointestinal” or “hematologic.” General gastrointestinal AEs were the most commonly reported; 221 of 642 patients assessed for AEs in clinical trials, across all severity grades, had gastrointestinal-related issues (not including nausea): 36.1% with MTX, and 39.7% with Act-D. Additionally, 115 patients experienced fatigue (MTX: 27.4%; Act-D: 29.1%), 187 had hematologic issues (MTX: 7.2%; Act-D: 8.0%), and 29 experienced alopecia (MTX: 5.8%; Act-D: 7.0%) (8–10, 37, 41, 43, 44, 55–57). More than one-fifth of patients reported the specific side effect of nausea (20.4%), but not every trial reported nausea as a separate AE.

Two interventional studies reported occurrences of diarrhea among 14 of 106 patients (13.2%) treated with MTX (37, 55) and three studies reported occurrences of diarrhea among 15 of 139 patients (10.8%) treated with Act-D (37, 44, 55). Some trials also reported detailed information about hematologic AEs. In three studies comparing Act-D (n=99) and MTX (n=133), 6.0% and 6.9% of patients receiving Act-D and MTX reported neutropenia as an AE, respectively (37, 55, 58). In one trial comparing EMA-CO (n=46) and the multiagent regimen of floxuridine, Act-D, etoposide, and vincristine (FAEV; n=43) in first-line GTN treatment, 32.6% patients receiving EMA-CO, and 60.1% patients receiving FAEV experienced grade 4 myelosuppression; also, 60.1% of patients treated with EMA-CO and 25.6% treated with FAEV experienced grade 4 neutropenia (43).

#### 3.9.2 AEs from observational studies

A total of 14 observational studies reported AEs, of which six specified the grade of AE (**Figure 3**). The specificity of the reported AEs varied considerably among observational studies. Among the 14 observational studies that reported AEs, 102 patients reported hair loss, 144 reported gastrointestinal-related effects, and 615 reported hematologic-related AEs (22, 27, 42, 59–68). Nausea was considered separately from these broad categories and was reported in 124 of 1,227 observed patients (10.1%).

**Figure 3:**
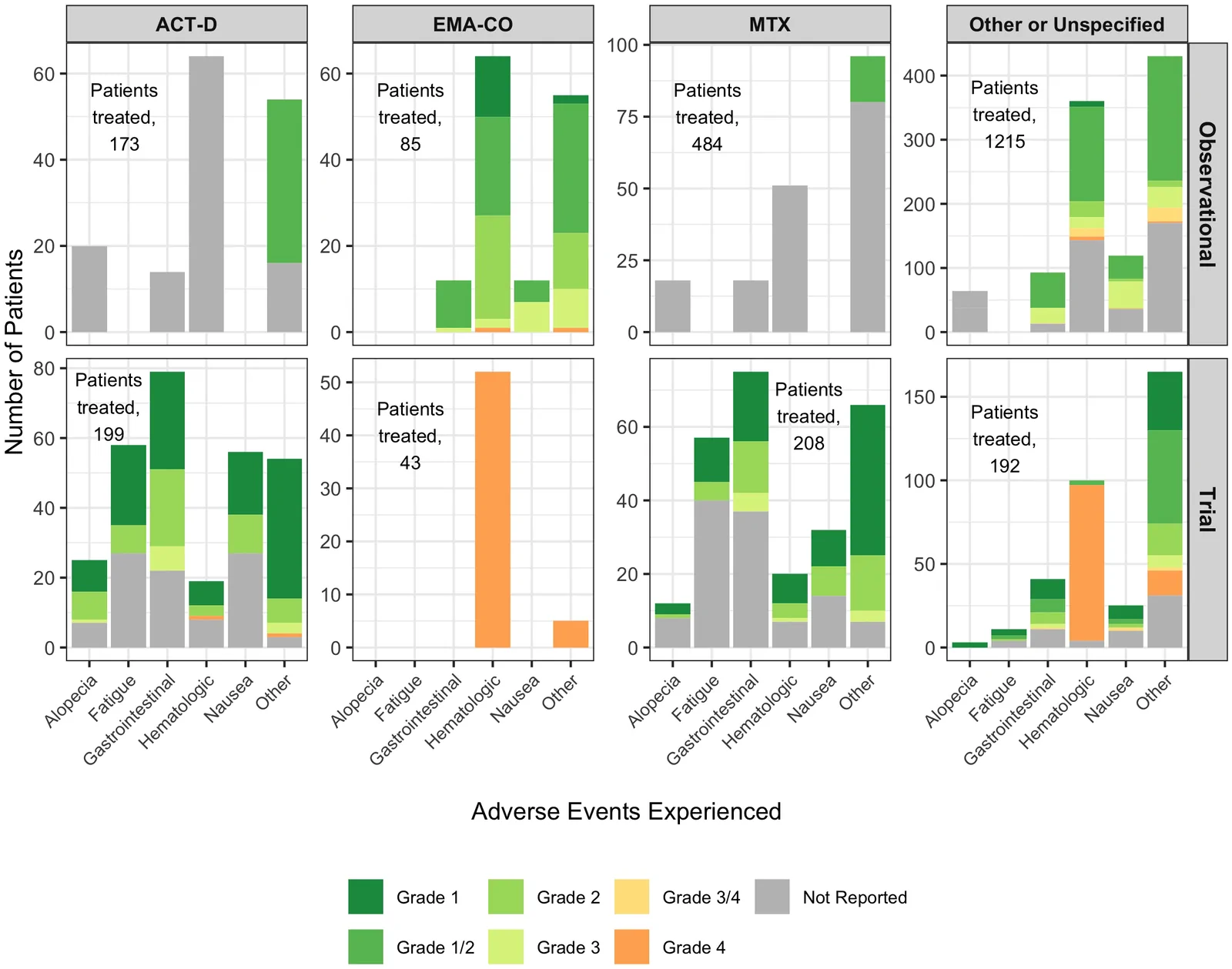
Reported adverse events in interventional and observational studies on GTN. Act-D, dactinomycin; EMA-CO, etoposide, MTX, Act-D, cyclophosphamide, and vincristine; GTN, gestational trophoblastic neoplasia; MTX, methotrexate.

The percentage of patients who received Act-D, EMA-CO, MTX, or other regimens and experienced alopecia by grade of toxicity is reported in **Figure 3**. Given the wide range of studies across various treatment lines and risk categories, it is challenging to attribute specific AEs by treatment. However, four studies reported leukopenia in 185 of 354 patients (52.2%) treated with single- and/or multiagent chemotherapy (22, 60, 62, 66). Similarly, two studies reported that 88 of 124 patients (71.0%) experienced neutropenia during treatment with a multiagent chemotherapy (27, 69).

### 3.10 Study quality

The methodological quality of included studies varied, reflecting the diverse study designs incorporated in this review (**Supplementary Figure 3**). A total of 87 studies were assessed using the Newcastle Ottawa Scale, including 86 cohort studies and one case-control study. The final graded scores ranged from 0.33 to 1.00, with a mean score of 0.78. A total of 21.9% of studies received a perfect score of 1.00, suggesting optimal methodological quality regarding selection, comparability, and outcomes. Nearly one-third (31.0%) were found to have moderate to high methodological quality, while 40.2% were deemed to have moderate methodological quality. In contrast, 6.8% of studies were found to have low methodological quality. The majority of studies demonstrated a clear definition of exposure and outcomes, with numerous studies featuring adequate follow-up periods. Nevertheless, a recurrent challenge was the inability to ensure comparability between groups due to an inadequate adjustment for confounders.

Two cross-sectional studies were evaluated using the National Heart, Lung, and Blood Institute Quality Assessment Tool. The total scores were 0.47 and 0.58, reflecting low to moderate methodological quality. Both studies had clearly defined objectives and populations, but shortcomings included unclear recruitment methods, low response rates, and limited consideration of confounding variables.

Among the 14 clinical trials assessed using the Risk of Bias in Non-Randomized Studies – of Interventions tool, 35.7% were classified as having a moderate risk of bias (moderate quality), 28.6% as low risk (high quality), 21.4% as low to moderate risk (moderate to high quality), and 14.3% as serious risk (low quality). The issues commonly identified included lack of randomization, residual confounding, and incomplete outcome data. However, most studies described interventions and outcomes clearly, with consistent protocols.

A total of eight SLRs were evaluated using the Assessing the Methodological Quality of Systematic Reviews version 2 tool​. The final scores ranged from 0.385 to 0.813 (mean: 0.625), indicating that the included SLRs were generally of moderate quality. While most reviews exhibited clearly defined objectives and comprehensive literature searches, commonly identified weaknesses included an absence of protocol registration, insufficient assessment of publication bias, and inadequate integration of risk of bias evaluation into synthesis.

## 4 Discussion

A total of 117 studies were included in this review, which covered epidemiological outcomes, treatment patterns, various clinical outcomes such as treatment response, and information on patient journey and related outcomes in GTN. Cohort studies comprised over two-thirds (66.7%) of all studies in the review. More than half of all studies (n=64; 54.7%) were conducted in Asia, and 13 studies (11.1%) were multiregional.

Incidence estimates of GTN varied widely by region, among other factors. For example, the incidence of GTN among >10,000 patients with GTD in the UK was 6.2% (27). while across 1,342 patients with GTD in nine Asian studies, the incidence of GTN was 16.8% (24, 26).

Among first-line regimens in observational studies, MTX was most commonly reported, followed by Act-D. However, large differences in study design and patient samples and a lack of standardization of regimens were observed, limiting summarization. Patients experiencing first-line treatment failure with single-agent chemotherapy were often treated by switching to either Act-D or MTX (from initial MTX or Act-D) or to a multiagent chemotherapy regimen such as EMA-CO. The overall success of single-agent therapy across multiple treatment lines in low-risk GTN is difficult to characterize across the diverse study designs covered in the review, but it should nonetheless be noted. Patients with low-risk GTN who receive salvage MTX or Act-D treatment following initial treatment have a 90% to 95% remission rate (7, 70).

However, treatment regimens for patients with high-risk disease were relatively diverse compared with the consensus associated with single-agent first-line therapy for low-risk GTN. The observed heterogeneity in treatments for high-risk patients was supported by Albright et al, who conducted an SLR and meta-analysis of the treatments and outcomes of patients with high-risk GTN (71). While ultra-high–risk individuals were not explicitly identified as a subgroup in this review, this group of patients, who generally receive etoposide-cisplatin induction with EMA-CO or other multiagent chemotherapy (72), represent those with the highest risk for poor outcomes, including death (71, 73).

The percentage of patients in clinical trials who achieved a measure of treatment response following first-line Act-D regimens (82.9%) appeared higher than the respective percentage among patients who received first-line MTX regimens (70.5%). Hao et al. reported similar findings in an SLR and meta-analysis of randomized and high-quality nonrandomized studies, noting that Act-D was superior to MTX in achieving complete remission (80.2% [551/687] vs 65.1% [643/987]), respectively) in both clinical trials and observational studies (63). Other reviews and meta-analyses comparing the efficacy of first-line single-agent chemotherapy in patients with low-risk GTN found Act-D more effective than MTX treatment, but these results varied at the study level and on the regimen dose and timing (69, 74). However, many studies that compared the effectiveness of treatments (63), including ours, do not focus on the differences in outcomes between dose regimens of the same primary drug.

Beyond Act-D and MTX, a network meta-analysis by Zhou and Kemin (69) found that a 5-day regimen of VP-16 (etoposide injection) was the most effective low-risk first-line monotherapy in a retrospective study from Japan in 1998 (75). However, this single study was outside the timeframe of our review and was therefore not included in this SLR.

In real-world settings, treatment response appeared to be associated with GTN risk score, with patients at higher risk being less likely to respond to both first- and second-line regimens. Across all 17 studies reporting complete response (across all therapy lines, agents, and risk categories), 77.0% of patients achieved complete response to treatment.

Across all lines of treatment, agents, and GTN risk categories, a mean of 27.8% (SD: 17.2%) of patients developed some form of resistance to chemotherapy; data indicated that a higher percentage of patients treated with MTX appeared to develop resistance (35.2%) vs those treated with Act-D (23.3%). Similarly, the mean percentage of patients who experienced recurrence appeared higher among those treated with MTX (9.1%) than Act-D (4.9%). Mortality was reported more rarely in studies and was not observed among low-risk patients treated with first-line Act-D; however, 5.9% of high-risk patients died following Act-D treatment.

The most common AEs reported in clinical trials and observational studies included gastrointestinal AEs, hematologic AEs, alopecia, and fatigue. In Act-D and MTX trials, gastrointestinal AEs were most common; however, when grade of AEs was reported, most were lower in severity (i.e., grade 1 or 2). In the present review, Act-D was associated with a higher incidence of nausea and other gastrointestinal AEs in both interventional and observational studies. This evidence was supported by a meta-analysis performed by Hao et al. (63) that also observed an increased risk of nausea and vomiting associated with Act-D compared with MTX. One trial comparing the efficacy of EMA-CO and FAEV reported AEs among participants; in both treatment arms, hematologic-related AEs were more common in frequency and severity than other trials involving Act-D and MTX (43).

In observational studies, patients receiving both fluorouracil and EMA-CO more commonly experienced AEs (particularly hematologic AEs, which were reported in nearly all patients) compared with those receiving Act-D or MTX (22, 27, 59). While the number of AEs per patient was higher with these therapies, they were most often reported as being low grade. Given the infrequent reporting of AE grade with Act-D and MTX treatments in observational studies, it is not possible to compare the severity of observed AEs between these treatments.

It is also difficult to explicitly compare the specific frequencies of AEs reported within studies due to the heterogeneity in AE reporting. For example, studies detailing the number of patients who experienced specific gastrointestinal AEs may have counted patients more than once if they experienced more than one type of gastrointestinal AE (22, 63); however, in studies reporting gastrointestinal disorders categorically, patients experiencing multiple gastrointestinal AEs were counted once (62, 66). Therefore, to best summarize the AE outcomes as identified in the reviewed literature, we chose to report the results summarized in the most commonly reported groups, including alopecia, fatigue, nausea, hematologic, gastrointestinal, and other AEs.

Regarding the patient journey, several studies reported on the importance of access to specialized care; for example, the shorter the distance to a GTN treatment center, the greater the likelihood of early diagnosis and successful treatment. These findings are supported by treatment guidelines issued by the Society of Obstetrician and Gynaecologists of Canada (54). Across the five guidelines included in the review, the recommended first-line therapy for low-risk GTN was consistently single-agent chemotherapy (MTX or Act-D, when specified) (53, 54, 76–78). The EMA-CO multiagent regimen was most frequently recommended as first-line therapy for patients with high-risk disease. However, treatment recommendations were generally more heterogeneous for clinical situations, including the treatment of patients with high-risk disease and second-line therapy. Outside the scope of this literature review, which included publications through January 2025, the European Organisation for the Treatment of Trophoblastic Disease (EOTTD), ESGO, Gynecologic Cancer InterGroup (GCIG), and ISSTD published global consensus guidelines in June 2025 for the treatment of GTD (73). Similar to the guidelines included in this systematic review, these new global guidelines recommend MTX or Act-D for low-risk, first-line treatment of GTN (73). The EOTTD-ESGO-GCIG-ISSTD guidelines also provide global consensus among high- and ultra-high–risk patients, who should be treated with multiagent chemotherapy in a GTD reference center (73).

The heterogeneity of study designs, study populations and subgroup analyses, outcome definitions, study quality, and other factors limited the ability to synthesize and interpret data in certain respects. For example, the feasibility of conducting a meta-analysis was evaluated for overall response (OR) and complete response (CR) reported from clinical trials (MTX OR in 2 trials, Act-D OR in 3 trials; MTX CR in 4 trials, Act-D CR in 3 trials). However, there were not enough studies reporting the same drug-specific outcome to statistically estimate heterogeneity for OR, conduct a subgroup or meta-regression for either outcome, or conduct a publication bias assessment for either outcome. While there is general consensus around the use of Act-D or MTX in first-line treatment of low-risk GTN, unmet needs still exist for these patients. For example, although Act-D was associated with better clinical outcomes such as complete remission vs MTX, Act-D was also associated with an increased risk of gastrointestinal AEs such as nausea and vomiting. MTX was also associated with a higher incidence of chemoresistance and GTN recurrence than Act-D. Positive clinical outcomes such as complete remission are still suboptimal for certain patients, including those with higher-risk disease. EMA-CO is often prescribed in this setting but also has a more severe AE profile, particularly with regard to hematologic AEs. The treatment landscape for second-line treatment and high-risk patients across all therapy lines was generally diverse (including both surgery and pharmacologic therapy).

Recent treatment guidelines have noted the potential use of immunotherapy for these patients but caution that more long-term data on the effects of these therapies are needed. Avelumab monotherapy showed modest activity after single-agent resistance and minimal efficacy post-multiagent chemotherapy (TROPHIMMUN), suggesting greater benefit earlier or in combination with other agents (9). Moreover, pembrolizumab and camrelizumab plus apatinib have also shown activity in multiagent-resistant or high-risk relapsed disease, supporting the role of PD-1/PD-L1 blockade in selected patients (10, 11). The results of the TROPHAMET 2 trial, in which 96.2% of low-risk patients achieved hCG normalization, highlight the possible role avelumab with MTX may play in increasing overall efficacy, safety, and tolerability of treatment for patients with low-risk GTN (8). As of March 2025, many additional clinical trials assessing immunotherapy use for GTN patients are recruiting or in progress (12).

### 4.1 Limitations

The SLR was designed as a review of current publications to better understand current information regarding the epidemiology and treatment of GTN. As such, robust quantitative analyses were outside the scope of this review. Moreover, given the wide range of studies included, reported outcomes varied and further limit the specificity of results presented. The limitations of the review are subject to the selection criteria for studies included. Relevant information may have therefore been excluded based on the search criteria (e.g., evidence published in articles in languages other than English). A risk of bias assessment indicated that study quality varied widely.

This SLR was global in nature and broad in scope. However, the generalizability of individual study results is dependent on the design and selection criteria used, which varied widely among included publications. For example, some studies reported national-level data, while others reported data from single institutions. Care must be taken when interpreting results across studies.

The study was not prospectively registered; however, a pre-specified study protocol guided the review and all methods are transparently described in full here. All anticipated limitations and biases are described above.

## 5 Conclusions

This SLR identified current data on the epidemiology, treatment patterns, clinical outcomes, patient journey, and therapies recommended by professional guidelines for the treatment of GTN. Over half of the studies were conducted in Asia, highlighting the need for evidence from other regions. While the studies included were diverse across factors such as study design, study populations, and outcomes, there is general consensus in the use of MTX or Act-D as first-line therapy in low-risk GTN. However, these regimens are not interchangeable, and unmet needs exist; for example, a greater likelihood of outcomes such as complete remission were observed with Act-D therapy vs MTX, although Act-D was associated with more gastrointestinal AEs. Among high-risk patients, EMA-CO was often used but was associated with tolerability issues, primarily hematologic AEs. Treatment recommendations most often differed among patients with high-risk disease and in the second line, although the most recent June 2025 EOTTD-ESGO-GCIG-ISSTD guidelines provide consensus that high- and ultra-high–risk patients should be treated with multiagent chemotherapy in a GTD reference center. Future analyses should indicate whether real-world treatment patterns follow these new global guidelines. Immunotherapy agents were reported by recent clinical guidelines as potential options for patients who experienced treatment failure with first-line therapy or for high-risk patients, but more research is warranted to investigate upfront immunotherapy combination therapy vs the current standard of care of single-agent chemotherapy.

## Data Availability

Publicly available datasets were analyzed in this study and this study was based on previously published data. Specific extracted data used in this review may be available upon request. The study was not registered in a public database prior to conducting the study; the study protocol may be available upon request. Any requests for data by qualified scientific and medical researchers for legitimate research purposes will be subject to Merck’s Data Sharing Policy. All requests should be submitted in writing to Merck’s data sharing portal (https://www.merckgroup.com/en/research/our-approach-to-research-and-development/healthcare/clinical-trials/commitment-responsible-data-sharing.html).
