# Supplementary material for: Epidemiology, treatment patterns, and outcomes in gestational trophoblastic neoplasia: a systematic review
Source: Front Oncol. 2026 Oct 14;16:1923113. doi: 10.3389/fonc.2026.1923113 (PMC13713317; doi:10.3389/fonc.2026.1923113)
Supplement: Supplementary file 1 [file DataSheet1.docx]

Supplementary Material

Tables

Table S1. PICOS-T criteria for the inclusion and exclusion of studies.

|  | **Inclusion criteria** | **Exclusion criteria** |
| --- | --- | --- |
| Population | GTN, including IM, CA, PSTT, and ETT | Non-human, animal |
| Intervention | No restrictions | NA |
| Comparator | No restrictions | NA |
| Outcome | Epidemiology, treatment patterns, outcomes with treatment (response rate, subsequent pregnancies, efficacy), hCG normalization rate, guidelines, approved therapies, unmet needs, patient journey | NA |
| Study Design | Observational studies, treatment guidelines*, interventional trials—single arm or randomized of any phase, SLRs**, case series***  English language only | In vitro/vivo, case reports, case series |
| Time | Published studies: January 2005 – January 2025; Conference abstracts January 2022 – January 2025 | Studies published before January 2005 (observational studies before January 2015), abstracts before January 2022 |

CA, choriocarcinoma; ETT, epithelioid trophoblastic tumor; GTN, gestational trophoblastic neoplasia; hCG, human chorionic gonadotropin; IM, invasive mole; NA, not applicable; PSTT, placental-site trophoblastic tumor.

*Only the most recent treatment guideline per issuing society was included in the review.

**SLRs primarily focused on publications in the specified timeframe of this study.

***Case series with more than 10 patients.

**Table S2.** List of included studies.

| **1** | Abu-Rustum NR, Yashar CM, Bean S, et al. (2019). Gestational trophoblastic neoplasia, version 2.2019, NCCN clinical practice guidelines in oncology. *Journal of the National Comprehensive Cancer Network*, 17, 1374-1391. |
| --- | --- |
| **2** | Agbari V, Hidayat YM, Kurniadi A, et al. (2024). Effectiveness and efficiency comparison of one-day vs eight-day methotrexate protocols in managing low-risk gestational trophoblastic neoplasia. *International Journal of Women's Health,* 16, 2077-2085. |
| **3** | Akgöl S, Oğlak SC, Tunç Ş, et al. (2022). Eight-day methotrexate/folinic acid regime as single agent chemotherapy for low-risk gestational trophoblastic neoplasia: a retrospective study. *Journal of Clinical Obstetrics & Gynecology,* 32(1), 1-6. |
| **4** | Akhavan S, Hoorshad N, Mousavi As, et al. (2024). The role of single-dose prophylactic methotrexate in the prevention of post-molar gestational trophoblastic neoplasia in patients with high-risk molar pregnancy. *BMC Cancer,* 24, 1400. |
| **5** | Albright BB, Ellett T, Knochenhauer HE, et al. (2023). Treatments and outcomes in high‐risk gestational trophoblastic neoplasia: a systematic review and meta‐analysis. *BJOG: An International Journal of Obstetrics & Gynaecology*, 130(5), 443-453. |
| **6** | Almohammadi NH. (2022). A histopathological profile of gestational trophoblastic disease in the Madinah Region of Saudi Arabia: a single institute experience. *Nigerian Journal of Clinical Practice,* 25, 1256-1261. |
| **7** | Alshwayyat S, Haddadin Z, Alshwayyat M, et al. (2024). Machine learning-driven survival prediction in gestational trophoblastic neoplasms: a focus on PSTT and ETT prognosis. *Frontiers in Oncology,* 14, 1457531. |
| **8** | Antaratani RC, Shruthi M. Study of gestational trophoblastic diseases at a tertiary care hospital in India. International Journal of Reproduction, Contraception, Obstetrics and Gynecology. 2018 Apr 1;7(4):1622-8. |
| **9** | Bangash AG, Tabassum S, Afridi F, et al. (2022). Pregnancy outcomes after chemotherapy for gestational trophoblast neoplasia. *Journal of Medical Sciences*, 30, 117-120. |
| **10** | Bolze P, Schoenen S, Margaillan M, et al. (2024). Chemotherapy is not needed when complete evacuation of gestational choriocarcinoma leads to hCG normalization. *European Journal of Surgical Oncology*, 50, 108012. |
| **11** | Braga A, Balthar E, Souza LCS, et al. (2023). Immunotherapy in the treatment of chemoresistant gestational trophoblastic neoplasia-systematic review with a presentation of the first 4 Brazilian cases. *Clinics*. 78, 100260. |
| **12** | Braga A, Canelas AC, Torres B, et al. (2022). Neutrophil/lymphocyte ratio and other blood cell component counts are not associated with the development of postmolar gestational trophoblastic neoplasia. *PLOS One.* 17(12), e0277892. |
| **13** | Braga A, Lopes R, Campos V, et al. (2023). The impact of the distance traveled between residence and gestational trophoblastic neoplasia reference center and clinical outcomes in Brazilian women. *Gynecologic Oncology*, 176, 130-138. |
| **14** | Braga A, Paiva G, Ghorani E, et al. (2021). Predictors for single-agent resistance in FIGO score 5 or 6 gestational trophoblastic neoplasia: a multicentre, retrospective, cohort study. *The Lancet Oncology,* 22, 1188-1198. |
| **15** | Campos V, Paiva G, Padron L, et al. (2023). Influence of COVID‐19 pandemic on molar pregnancy and postmolar gestational trophoblastic neoplasia: an observational study. *BJOG*, 130(3), 292-302. |
| **16** | Capobianco G, Tinacci E, Saderi L, et al. (2021). High incidence of gestational trophoblastic disease in a third-level university-hospital, Italy: a retrospective cohort study. *Frontiers in Oncology*, 11, 684700. |
| **17** | Chadha S, Gupta S, Kalwaniya DS, et al. (2024). Navigating gestational trophoblastic neoplasia: lessons from 15 cases at a North Indian tertiary care center. *Indian Journal of Gynecologic Oncology*, 22, 157. |
| **18** | Chambers LM, Chalif J, Vargas R. (2022). Analysis of patient experiences with gestational trophoblastic neoplasia reported on Instagram social media. *Gynecologic Oncology*, 165(3), 603-609. |
| **19** | Chen D, Yang Y, Li Y, et al. (2022). 5 Fluorouracil as first-line treatment for low-risk gestational trophoblastic neoplasia. *Tropical Journal of Pharmaceutical Research*, 21, 2669-2676. |
| **20** | Cheng H, Zong L, Kong Y, et al. (2021). Camrelizumab plus apatinib in patients with high-risk chemorefractory or relapsed gestational trophoblastic neoplasia (CAP 01): a single-arm, open-label, phase 2 trial. *The Lancet Oncology,* 22, 1609-1617. |
| **21** | Covens A, Filiaci VL, Burger RA, et al. (2006). Phase II trial of pulse dactinomycin as salvage therapy for failed low‐risk gestational trophoblastic neoplasia: a Gynecologic Oncology Group study. *Cancer,* 107(6), 1280-1286. |
| **22** | Descargues P, Hajri T, Massardier J, et al. (2021). Gestational trophoblastic neoplasia after human chorionic gonadotropin normalization in a retrospective cohort of 7761 patients in France. *American Journal of Obstetrics and Gynecology*, 225(4), 401.e1-401.e9. |
| **23** | Devi LP, Devi AA, Kamei R, et al. (2023). Patterns of gynaecological malignancies in a tertiary care hospital in North East India. *Research Journal of Medical Science*, 17, 171-174. |
| **24** | Diver E, Richardson M, Liao CI, et al. (2021). Age and racial differences in the presentation of gestational trophoblastic neoplasia. *International Journal of Gynecological Cancer*, 31, 194-202. |
| **25** | Edesa WA, Ayad NN, Mounir AM, et al. (2022). Treatment outcome of gestational trophoblastic neoplasia patients in Egypt. *Indian Journal of Cancer*, 59(1), 46-53. |
| **26** | Eiriksson L, Dean E, Sebastianelli A, et al. (2021). Guideline No. 408: management of gestational trophoblastic diseases. *Journal of Obstetrics and Gynaecology Canada*, 43, 91-105. |
| **27** | Fehlmann A, Benkortbi K, Rosseel G, et al. (2021). Gestational trophoblastic disease in Switzerland: retrospective study of the impact of a regional reference centre. *Swiss Medical Weekly*, 151, w20406. |
| **28** | Fülöp V, Szigetvári I, Szepesi J, et al. (2021). The diagnostics and treatment of low-risk gestational trophoblastic neoplasia (GTN): 42-year experience. *European Journal of Gynaecological Oncology*, 42(6). |
| **29** | Galea M, Descargues P, Hajri T, et al. (2025). Severe uterine haemorrhagic complications from gestational trophoblastic neoplasia. *European Journal of Obstetrics & Gynecology and Reproductive Biology*, 304, 30-34. |
| **30** | Ghosh J, Dey S, Mandal D, et al. (2021). Clinicopathological features and outcomes of choriocarcinoma: a retrospective analysis from an Indian tertiary cancer center. *Cancer Research, Statistics, and Treatment*, 4, 486-491. |
| **31** | Gilani MM, Yarandi F, Eftekhar Z, et al. (2005). Comparison of pulse methotrexate and pulse dactinomycin in the treatment of low‐risk gestational trophoblastic neoplasia. *Australian and New Zealand Journal of Obstetrics and Gynaecology*, 45, 161-164. |
| **32** | Gorun F, Tomescu L, Motoc A, et al. (2022). Clinical features and management of trophoblastic epithelioid tumors: a systematic review. *Medicine*, 101, e29934. |
| **33** | Gu Y, Liu Y, Cheng H, et al. (2024). Clinicopathological characteristics and oncologic outcomes of patients with gestational trophoblastic neoplasia manifesting as isolated pulmonary lesions. *Gynecologic Oncology*, 190, 28-34. |
| **34** | Hajri T, Massoud M, Vergne M, et al. (2024). Multiple pregnancy with complete hydatidiform mole and coexisting normal fetus in a retrospective cohort of 141 patients. *American Journal of Obstetrics and Gynecology*, 230, 362.e1-362.e8. |
| **35** | Hao J, Zhou W, Zhang M, et al. (2021). Direct comparisons of efficacy and safety between actinomycin-D and methotrexate in women with low-risk gestational trophoblastic neoplasia: a meta-analysis of randomized and high-quality non-randomized studies. *BMC Cancer*, 21, 1-14. |
| **36** | Hasanzadeh M, Homaee Shandiz F, Izadpanahee N, et al. (2022). Is there a role for surgery in the management of gestational trophoblastic neoplasia? *Middle East Journal of Cancer,* 13, 128-134. |
| **37** | Hassan AR, Itsura PM, Rosen BP, et al. (2024). Mortality factors in high and ultra-high-risk gestational trophoblastic neoplasia at moi teaching & referral hospital: a decade-long observation in Kenya. *Gynecologic Oncology Reports,* 53, 101392. |
| **38** | Horowitz NS, Eskander RN, Adelman MR, Burke W. Epidemiology, diagnosis, and treatment of gestational trophoblastic disease: A Society of Gynecologic Oncology evidenced-based review and recommendation. Gynecologic Oncology. 2021 Dec 1;163(3):605-13. |
| **39** | Hou YM, Li PP, Yu H, et al. (2024). Clinical features and demographic characteristics of gestational trophoblastic neoplasia: single center experience and the SEER database. *Biomolecules and Biomedicine*, 24, 176. |
| **40** | Hussain SS, Raees M, Rahim R. (2022). Ten-year review of gestational trophoblastic disease at Lady Reading Hospital, Peshawar. *Cureus*, 14, e26620. |
| **41** | Jareemit N, Therasakvichya S, Freitas F, et al. (2023). EMACO for treatment of gestational trophoblastic neoplasia: a multinational multicenter study. *Gynecologic Oncology*, 170, 114-122. |
| **42** | Ji M, Jiang S, Zhao J, et al. (2022). Efficacies of FAEV and EMA/CO regimens as primary treatment for gestational trophoblastic neoplasia. *British Journal of Cancer*, 127, 524-530. |
| **43** | Jiang F, Lin JK, Xiang Y, et al. (2022). The impact of pulmonary metastases on therapeutic response and prognosis in malignant gestational trophoblastic neoplasia patients: a retrospective cohort study. *European Journal of Cancer*, 161, 119-127. |
| **44** | Jiang S, Zhao J, Shi X, et al. (2023). Retrospective analysis of clinical features and fertility outcomes with fertility-sparing treatment of placental site trophoblastic tumor. *Gynecologic Oncology*, 171, 1-8. |
| **45** | Jiao L, Wang Y, Jiang J, et al. (2022). Centralized surveillance of hydatidiform mole: 7-year experience from a regional hospital in China. *International Journal of Gynecological Cancer*, 32(2), 147-152. |
| **46** | Jin T, Zhou Z, Lin M, et al. (2024). Risk factors for methotrexate resistance in low-risk gestational trophoblastic neoplasia patients (FIGO score 0-4). *American Journal of Cancer Research*, 14, 1353. |
| **47** | Kale İ, Topal CS. (2021). Evaluation of gestational trophoblastic diseases; 10 years’ experience in tertiary obstetric care center. *Zeynep Kamil Medical Journal*, 52, 170-176. |
| **48** | Kamatham V, Dorairajan J, Keepanasseril A. (2024). Clinical profile of hydatidiform mole-a cohort study. *International Journal of Medicine & Public Health*, 14, 55-61. |
| **49** | Kang HL, Zhao Q, Yang SL, et al. (2019). Efficacy of combination therapy with actinomycin D and methotrexate in the treatment of low-risk gestational trophoblastic neoplasia. *Chemotherapy*, 64, 42-47. |
| **50** | Kemin L, Mengpei Z, Rutie Y. (2022). Monotherapy for low-risk gestational trophoblastic neoplasia with score 5-6. *Frontiers in Oncology*, 12, 1035170. |
| **51** | Kong Y, Wang W, Lin J, et al. (2022). Management and predictors of treatment failure in patients with chemo-resistant/relapsed gestational trophoblastic neoplasia with lung metastasis. *Journal of Clinical Medicine*, 11, 7270. |
| **52** | Kurniadi A, Zulvayanti Z, Suardi D, et al. (2022). Risk factors that correlate with resistance to first-line chemotherapy on high-risk gestational trophoblastic neoplasia. *Open Access Macedonian Journal of Medical Sciences*, 10, 1446-1449. |
| **53** | Lai THT, Lau LSK, Ngu SF, et al. (2024). Comparison of the multiples of the median of serum anti‐müllerian hormone and pregnancy outcomes in patients with gestational trophoblastic disease: a case–control study. *Cancer Medicine*, 13, e7134. |
| **54** | Lertkhachonsuk AA, Israngura N, Wilailak S, et al. (2009). Actinomycin d versus methotrexate-folinic acid as the treatment of stage I, low-risk gestational trophoblastic neoplasia: a randomized controlled trial. *International Journal of Gynecological Cancer*, 19, 985-988. |
| **55** | Li Y, Chen D, Yu H, et al. (2022). The efficacy evaluation of initial chemotherapy for high-risk gestational trophoblastic neoplasm. *Current Problems in Cancer*, 46, 100861. |
| **56** | Liang H, Pan NP, Wang YF, et al. (2022). Obstetrical and oncological outcomes of twin pregnancies with hydatidiform mole and coexisting fetus. *Taiwanese Journal of Obstetrics and Gynecology*, 61(3), 453-458. |
| **57** | Liu C, Ouyang P, Tao Y, et al. (2024). Analysis of the efficacy of 5-fluorouracil in the treatment of invasive moles. *Clinical and Experimental Obstetrics & Gynecology*, 51, 83. |
| **58** | Liu W, Zhao W, Huang X. (2022). Outcomes and prognostic factors of placental-site trophoblastic tumor: a retrospective study of 58 cases. *Archives of Gynecology and Obstetrics*, 306, 1633-1641. |
| **59** | Liu W, Zhou J, Yang J, et al. (2022). A multicenter retrospective study of epithelioid trophoblastic tumors to identify the outcomes, prognostic factors, and therapeutic strategies. *Frontiers in Oncology*, 12, 907045. |
| **60** | Liu YL, Praiss AM, Chiang S, et al. (2025). Gestational trophoblastic neoplasm: Patient outcomes and clinical pearls from a multidisciplinary referral center. *Gynecologic Oncology*, 192, 171-177. |
| **61** | Liu Y, Ye Y, Cheng X, et al. (2023). The effect of prophylactic chemotherapy on treatment outcome of postmolar gestational trophoblastic neoplasia. *BMC Women's Health*, 23, 1. |
| **62** | Madi JM, Paganella MP, Litvin IE, et al. (2022). Perinatal outcomes of first pregnancy after chemotherapy for gestational trophoblastic neoplasia: a systematic review of observational studies and meta-analysis. *American Journal of Obstetrics and Gynecology*, 226, 633-645. |
| **63** | McCullough L, Danihel L, Sufliarsky J, et al. (2024). Invasive and metastatic hydatidiform moles in Slovakia in 1993-2022. *Bratislava Medical Journal/Bratislavské Lekárske Listy*, 125:423-428. |
| **64** | McInerney C, McNally O, Cade TJ, et al. (2024). Low‐risk gestational trophoblastic neoplasia–20 years experience of a state registry. *Australian and New Zealand Journal of Obstetrics and Gynaecology*, 64(3), 223-229. |
| **65** | Mdoe MB, Mwakigonja AR, Mwampagatwa I. (2023). Gestational trophoblastic disease and associated factors among women experiencing first trimester pregnancy loss at a regional referral hospital in central Tanzania: a cross-sectional study. *International Health*, 15, 250-257. |
| **66** | Mitric C, Yang K, Bhat G, et al. (2023). Gestational trophoblastic neoplasia: does centralization of care impact clinical management? *International Journal of Gynecological Cancer*, 33, 1724-1732. |
| **67** | Mousavi A, Cheraghi F, Yarandi F, et al. (2012). Comparison of pulsed actinomycin D versus 5-day methotrexate for the treatment of low-risk gestational trophoblastic disease. *International Journal of Gynecology & Obstetrics*, 116, 39-42. |
| **68** | Munyakarama B, Koushik A, Leduc V, et al. (2025). Association of gestational trophoblastic disease with subsequent development of non‐trophoblastic cancer. *International Journal of Gynecology & Obstetrics,* 168, 1305-1311. |
| **69** | Muralidas A, Baby SA. (2022). Evaluation of incidence, risk factors, diagnosis and management of gestational trophoblastic neoplasia: a retrospective study. *Journal of Clinical & Diagnostic Research*, 16, QC14-QC18. |
| **70** | Mylvaganam G, Allanson E, Allanson B, et al. (2021). Assessment of current follow‐up for complete molar pregnancies: a single centre review. *Australian and New Zealand Journal of Obstetrics and Gynaecology*, 61, 213-216. |
| **71** | Nguyen BQ, Vo TM, Nguyen C, et al. (2023). Clinical features of gestational trophoblastic disease in aged women in South Vietnam. *Yonsei Medical Journal*, 64, 284. |
| **72** | Niharika D, Mallipeddi S, Sweta B. (2023). Gynaecological cancers: an analysis of prevalence, demography and effect of multi-modality cancer treatment in a tertiary care hospital. *International Journal of Pharmaceutical and Clinical Research,* 15, 290-298. |
| **73** | Othman M, Hemida R, Foda A, et al. (2024). Upfront hysterectomy versus primary chemotherapy in the management of low-risk gestational trophoblastic neoplasia at old age patients: a prospective study. *Indian Journal of Gynecologic Oncology*, 22, 33. |
| **74** | Parker VL, Cushen BF, Hills A, et al. (2023). Flat-dose versus weight or body surface area-based methotrexate dosing in low-risk gestational trophoblastic neoplasia. *Gynecologic Oncology*, 169, 34-40. |
| **75** | Patel O, Pradhan S, Dora SK, et al. (2024). Clinicopathological study of gestational trophoblastic diseases. *Journal of Pharmaceutical Negative Results,* 13, 8964-8972. |
| **76** | Patel SP, Othus M, Chae YK, et al. (2024). A phase II basket trial of dual anti–CTLA-4 and anti–PD-1 blockade in rare tumors (DART SWOG 1609 cohort 47) in patients with gestational trophoblastic neoplasia. *Clinical Cancer Research*, 30, 33-38. |
| **77** | Peyle M, Massoud M, Patrier S, et al. (2024). Impact of molecular genotyping on the diagnosis and treatment of human chorionic gonadotropin-producing tumors. *Journal of Gynecology Obstetrics and Human Reproduction*, 53, 102704. |
| **78** | Phianpiset R, Ruengkhachorn I, Kuljarusnont S, et al. (2022). Predictive factors associated with resistance to initial methotrexate treatment in women with low‐risk gestational trophoblastic neoplasia. *Asia‐Pacific Journal of Clinical Oncology*, 18, e495-e506. |
| **79** | Piazzetta SR, Hoch KA, Benetti-Pinto CL, et al. (2024). Assessment of risk factors associated with post-molar gestational trophoblastic neoplasia: a retrospective cohort. *Revista Brasileira de Ginecologia e Obstetrícia*, 46, e-rbgo83. |
| **80** | Rakprasit C, Ruengkhachorn I, Therasakvichya S, et al. (2023). Combined analysis of clinical features, human chorionic gonadotropin (hCG) value, and hCG ratios for early prediction of postmolar gestational trophoblastic neoplasia. *Archives of Gynecology and Obstetrics*, 307, 1145-1154. |
| **81** | Ramesan CK, Thomas DS, Sebastian A, et al. (2021). Role of hysterectomy in gestational trophoblastic neoplasia. *Indian Journal of Surgical Oncology*, 12, 386-390. |
| **82** | Ramírez LAC, Maestá I, Bianconi MI, et al. (2022). Clinical presentation, treatment outcomes, and resistance-related factors in South American women with low-risk postmolar gestational trophoblastic neoplasia. *Revista Brasileira de Ginecologia e Obstetrícia*, 44, 746-754. |
| **83** | Rivera N, Reid AB, Phillis M, et al. (2022). Surveillance after human chorionic gonadotropin normalization following molar pregnancy: a systematic review and meta-analysis. *Journal of Gynecologic Surgery*, 38, 86-95. |
| **84** | Sahoo S, Palo I, Sahu M, et al. (2023). Clinicopathological study of gestational trophoblastic disease (GTD) in a tertiary care hospital. *Indian Journal of Gynecologic Oncology*, 21, 71. |
| **85** | Schink JC, Filiaci V, Huang HQ, et al. (2020). An international randomized phase III trial of pulse actinomycin-D versus multi-day methotrexate for the treatment of low risk gestational trophoblastic neoplasia; NRG/GOG 275. *Gynecologic Oncology*, 158, 354-360. |
| **86** | Seresht LM, Farazestanian M, Yousefi Z. (2023). Single-agent chemotherapy in low-risk gestational trophoblastic neoplasia. *Caspian Journal of Internal Medicine*, 14, 108. |
| **87** | Sheikhhasani S, Abdolrazaghnejad A, Mousavi AS, et al. (2023). Resistance to single-agent chemotherapy in low-risk gestational trophoblastic neoplasia. *Caspian Journal of Internal Medicine*, 14, 47. |
| **88** | Singh K, Gillett S, Ireson J, et al. (2021). M‐EA (methotrexate, etoposide, dactinomycin) and EMA‐CO (methotrexate, etoposide, dactinomycin/cyclophosphamide, vincristine) regimens as first‐line treatment of high‐risk gestational trophoblastic neoplasia. *International Journal of Cancer*, 148, 2335-2344. |
| **89** | Soumya BM, Rajalakshmi D, Kulkarni S, et al. (2022). Histomorphological analysis of gestational trophoblastic disease spectrum with clinicopathological correlation at a teaching hospital. *Acta Medica International*, 9, 147-152. |
| **90** | Sugrue R, Foley O, Elias KM, et al. (2021). Outcomes of minimally invasive versus open abdominal hysterectomy in patients with gestational trophoblastic disease. *Gynecologic Oncology*, 160, 445-449. |
| **91** | Tejas SV, Pallavi VR, Shobha K, et al. (2022). Prognostic factors associated with hCG remission and chemotherapy resistance in patients with high-risk gestational trophoblastic neoplasia. *Indian Journal of Gynecologic Oncology*, 20, 52. |
| **92** | Tejas SV, Pallavi VR, Shobha K, et al. (2022). Role of salvage surgery in gestational trophoblastic neoplasia: a regional cancer centre experience. *Indian Journal of Surgical Oncology,* 13, 702-706. |
| **93** | Tempfer C, Horn LC, Ackermann S, et al. (2023). Gestational and non-gestational trophoblastic neoplasia. Guideline of the DGGG, OEGGG and SGGG (S2k-Level, AWMF Registry No. 032/049, April 2022). *Geburtshilfe und Frauenheilkunde*, 83, 267-288. |
| **94** | Tidy J, Seckl M, Hancock BW, on behalf of the Royal College of Obstetricians and  Gynaecologists. Management of Gestational Trophoblastic Disease. BJOG 2021;128:e1–e27. |
| **95** | Uberti EMH, de Freitas Medeiros LR, Cardoso RB, et al. (2025). Primary prevention of post-molar gestational trophoblastic neoplasia in high-risk complete hydatidiform mole: a single-dose prophylactic actinomycin D, associated with uterine evacuation-a long retrospective cohort study. *Gynecologic Oncology*, 193, 105-112. |
| **96** | Usman AU, Bello SS, Abdurrahman A, et al. (2024). A histopathological review of gynaecological malignancies in Katsina state North-Western Nigeria. *ecancermedicalscience*, 18, 1750. |
| **97** | Vo T, Tran T, Ho T, et al. (2023). Clinical evaluation of hysterectomy for the treatment of invasive mole in Southern Vietnam. *European Review for Medical & Pharmacological Sciences*, 27, 7720-7727. |
| **98** | Wallin E, Niemann I, Faaborg L, et al. (2022). Differences in administration of methotrexate and impact on outcome in low-risk gestational trophoblastic neoplasia. *Cancers*, 14, 852. |
| **99** | Wang K, Chen Y. (2021). Management and prognostic analysis of patients with gestational trophoblastic neoplasia (GTN) in FIGO stage IV and its special type. *Clinical & Experimental Metastasis*, 38, 47-59. |
| **100** | Wang L, Wang Q, Xu Z, et al. (2023). The efficacy and safety of 5-fluorouracil/cisplatin/vincristine as a multi-agent chemotherapy regimen in gestational trophoblastic neoplasia. *Frontiers in Oncology*, 13, 1240972. |
| **101** | Wang M, Shen L, Xu X, et al. (2021). Real‐world study of cisplatin, etoposide, and bleomycin chemotherapy regimen in gestational trophoblastic neoplasia. *BioMed Research International*, 2021, 6661698. |
| **102** | Wang X, Cang W, Liu X, et al. (2023). Anti-PD-1 therapy plus chemotherapy versus anti-PD-1 therapy alone in patients with high-risk chemorefractory or relapsed gestational trophoblastic neoplasia: a multicenter, retrospective study. *EClinicalMedicine*, 59, 101974. |
| **103** | Wang X, Cheng Y, Li W, et al. (2022). Management of relapsed gestational trophoblastic neoplasia: what can help to reduce the re‐recurrence rate? *Journal of Obstetrics and Gynaecology Research*, 48, 1451-1457. |
| **104** | Welsh M, Morrison S, Baartz D, et al. (2025). Outcomes of patients registered with the Queensland Trophoblast Centre diagnosed with gestational trophoblastic neoplasia who develop resistance to chemotherapy. *Australian and New Zealand Journal of Obstetrics and Gynaecology,* 65, 398-403. |
| **105** | Winarno GNA, Mulyantari AI, Kurniadi A, et al. (2022). Predicting chemotherapy resistance in gestational trophoblastic neoplasia: ratio of neutrophils, lymphocytes, monocytes, and platelets. *Medical Science Monitor: International Medical Journal of Experimental and Clinical Research*, 28, e938499. |
| **106** | Winter MC, Tidy JA, Singh K, et al. (2023). Efficacy analysis of single-agent carboplatin AUC4 2-weekly as second-line therapy for methotrexate-resistant (MTX-R) low risk gestational trophoblastic neoplasia (GTN). *Gynecologic Oncology*, 175, 66-71. |
| **107** | Xu J, Wang X. (2022). Comparing and evaluating five-day chemotherapy agents actinomycin D and methotrexate in low-risk post-molar gestational trophoblastic neoplasia: a retrospective analysis. *Clinical and Experimental Obstetrics & Oncology,* 49, 106. |
| **108** | Yamagami W, Mikami M, Nagase S, et al. (2020). Japan Society of Gynecologic Oncology 2018 guidelines for treatment of uterine body neoplasms. *Journal of Gynecologic Oncology*, 31, e18. |
| **109** | Yamamoto E, Kanal K, Soeung SC, et al. (2021). The current situation of health services for hydatidiform mole in Cambodia. *Journal of Obstetrics and Gynaecology Research,* 47, 3652-3660. |
| **110** | Yamamoto E, Nishino K, Niimi K, et al. (2022). Epidemiologic study on gestational trophoblastic diseases in Japan. *Journal of Gynecologic Oncology,* 33, e72. |
| **111** | Yarandi F, Eftekhar Z, Shojaei H, et al. (2008). Pulse methotrexate versus pulse actinomycin D in the treatment of low-risk gestational trophoblastic neoplasia. *International Journal of Gynecology & Obstetrics*, 103, 33-37. |
| **112** | Yarandi F, Mohseni M, Alikhasi M, et al. (2021). Predictive value of B-human chorionic gonadotropin for progression of molar pregnancy to persistent gestational trophoblastic neoplasm. *Minerva Obstetrics and Gynecology*, 75, 205-212. |
| **113** | You B, Bolze PA, Lotz JP, et al. (2023). Avelumab in patients with gestational trophoblastic tumors with resistance to polychemotherapy: cohort B of the TROPHIMMUN phase 2 trial. *Gynecologic Oncology,* 168, 62-67. |
| **114** | You B, Lotz JP, Descargues P, et al. (2024). 711O Avelumab+ methotrexate to eradicate low-risk gestational trophoblastic tumors in first-line setting: TROPHAMET phase I/II trial. *Annals of Oncology*, 35(suppl 2), S545. |
| **115** | Yuan LJ, Chen YY, Zhu CX, et al. (2022). Misdiagnosis and chemotherapy delaying reduces the chemosensitivity of choriocarcinoma patient: analysis of 36 cases. *Clinical and Experimental Obstetrics & Gynecology,* 49, 179. |
| **116** | Zhang T, Guo Y, He X, et al. (2024). Effect of lung metastasis on the treatment and prognosis of patients with gestational trophoblastic neoplasia: a systematic review and meta‐analysis. *Acta Obstetricia et Gynecologica Scandinavica*, 103, 636-644. |
| **117** | Zhou F, Kemin L. (2024). First-line monodrug chemotherapy in low-risk gestational trophoblastic neoplasia: a network meta-analysis. *Frontiers in Oncology*, 13, 1276771. |

Table S3. Frequency of regimens used in clinical trials, by line of therapy and agents.

| **Line of treat-ment** | **Agents used** | **Specific treatment details** | **Studies** |
| --- | --- | --- | --- |
| First line | MTX | MTX 50 mg/m^2^ via IM injection | 1 [38] |
|  |  | MTX 30 mg/m^2^/weekly/IM | 2 [39, 40] |
|  |  | IM MTX 0.4 mg/kg/day for 5 consecutive days per course | 2 [41] |
|  |  | IM MTX 1 mg/kg per day on days 1, 3, 5, and 7, alternating with IM folinic acid 0.1 mg/kg per day on days 2, 4, 6, and 8 | 1 [42] |
|  |  | IM MTX 50 mg/day on days 1, 3, 5, and 7, alternating with IM folinic acid 15 mg/day on days 2, 4, 6, and 8. The course is repeated on day 14. | 1 [43] |
|  |  | Institutional preference of either IV MTX (0.4 mg/kg) daily for 5 days every 14 days with a maximum daily dose of 25 mg or IM MTX (50 mg) on days 1, 3, 5, and 7 (4 doses per cycle) with leucovorin (15 mg) orally on days 2, 4, 6, and 8 within 24–30 h of the preceding day's injection. This is repeated every 14 days | 1 [44] |
|  | MTX + avelumab | 8-day MTX regimen: MTX 1 mg/kg IM on days 1, 3, 5, and 7, alternating with oral folinic acid; Q2W avelumab: flat dose IV 800 mg Q2W on day 1 before MTX | 1 [45] |
|  | Hysterectomy + MTX | Hysterectomy first, then  IM MTX 50 mg/day on days 1, 3, 5, and 7, alternating with IM folinic acid 15 mg/day on days 2, 4, 6, and 8. The course is repeated on day 14 | 1 [43] |
|  | Act-D | 1.25 mg/m^2^/biweekly/IV Act-D | 2 [39] |
|  |  | IV infusion of Act-D 10 μg/kg/day in 500 mL 5% glucose for 5 days per course | 1 [46] |
|  |  | 10 mg/kg/day IV for 5 days | 1 [42] |
|  |  | IV bolus of 1.25mg/m^2^ actinomycin D | 1 [41] |
|  |  | IV pulse actinomycin-D (1.25 mg/m^2^) once every 14 days with a maximum dose of 2 mg | 2 [40, 44] |
|  | Act-D + MTX | IV infusion of Act-D at a reduced dose of 8 µg/kg/day in 500 mL 5% glucose for 5 days with IM injection of MTX at a fixed dose of 25 mg every other day for each course | 1 [46] |
|  | FAEV (high risk) | Day 1: vincristine 2 mg, intravenous bolus, 3 h before actinomycin D; days 1–5: Act-D 0.2 mg/m^2^/day, IV infusion for >30 min; etoposide 100 mg/m^2^/day, IV infusion for >30 min; and floxuridine 800–900 mg/m^2^/day, IV infusion for 6–8 h | 1 [47] |
|  | EMA-CO  (high risk) | EMA day 1: Act-D 0.5 mg, IV infusion for 1 h; etoposide 100 mg/m^2^, IV infusion for 1 h; MTX 100 mg/m^2^, IV bolus; MTX 200 mg/m^2^, IV infusion for 12 h; EMA day 2: actinomycin D 0.5 mg, IV infusion for 1 h; etoposide 100 mg/m^2^, IV infusion for 1 h; calcium folinate 15 mg, IM injection, every 12 h for four doses, starting 12 h after treatment with MTX. CO day 8: vincristine 2 mg, IV infusion, 3 h before cyclophosphamide; cyclophosphamide 600 mg/m^2^, IV infusion for 2 h | 1 [47] |
| Second line or later | Act-D | IV pulse Act-D (1.25 mg/m^2^) once every 14 days with a maximum dose of 2 mg (following failed first-line low-risk treatment) | 1 [48] |
|  | Camrelizumab and apatinib | Camrelizumab 200 mg IV every 2 weeks and apatinib 250 mg orally once per day | 1 [49] |
|  | Nivolumab and ipilimumab | Nivolumab 240 mg IV Q2W and ipilimumab 1 mg/kg IV every 6 weeks on a continuous schedule | 1 [50] |
| Act-D, actinomycin-D/dactinomycin; EMA, etoposide, methotrexate, and actinomycin-D; EMA/CO, floxuridine,  dactinomycin, and vincristine; etoposide, methotrexate, dactinomycin/cyclophosphamide, and vincristine; FAEV, floxuridine, dactinomycin, etoposide, and vincristine; IM, intramuscular; IV, intravenous; MTX, methotrexate; Q2W, every 2 weeks. | | | |

**Table S4.** Frequency of regimens used in observational studies, by line of therapy and agents.

| **Line of treatment** | **Primary chemotherapy agent** | **Regimen** | | **No. of Studies** | |
| --- | --- | --- | --- | --- | --- |
| First line | MTX | One-day MTX protocol (300 mg/m^2^ 6–12 h IV infusion with leucovorin 15 mg orally or IM every 12 hours for 4 doses start 24 hours after MTX infusion | | 1 (51) | |
|  |  | Eight-day MTX protocol (50 mg MTX IM on days 1, 3, 5, and 7 with folinic acid 6-15 mg orally 24 hours after MTX on days 2, 4, 6, and 8) | | 8 (29, 51–57) | |
|  |  | MTX 30 mg/m^2^/weekly/IM | | 1 (58) | |
|  |  | MTX 0.4 mg/kg per day, maximum 25 mg) injected IM or IV for 5 days every other week | | 5 (10, 18, 59–61) | |
|  |  | MTX 10 mg given orally days 1–5 every 2–4 weeks, adding up to 50 mg MTX per cycle | | 1 (57) | |
|  | Act-D | Act-D 0.5 mg IV for 5 days | | 2 (53, 61) | |
|  |  | 1.25 mg/m^2^/biweekly/IV slowly (maximum dose of 2 mg) | | 4 (10, 58, 62, 63) | |
|  | 5-FU | Single-agent first-line treatment regimen of IV 5‑FU for 8 days | | 2 (29, 64) | |
|  |  | 5-FU (0.25 g/tube) (daily dose of 26–27 mg/kg in 500 mL of 5% glucose, administered within 6–8 hours), for 5 consecutive days | | 1 (65) | |
| High risk or second line* | M-EA | GCSF (300 or 480 mcg according to weight SC daily for 5 days) was generally introduced as secondary prophylaxis following an episode of neutropenia (± treatment delay) or neutropenic sepsis | 1 (22) | |  |
|  | EMA-CO | Primary prophylactic GCSF (300 or 480 mcg according to weight SC daily for 5 days) following the CO only as per protocol. This protocol was later amended after 13 patients had received EMA-CO to titrating the GCSF according to the baseline neutrophil count. Patients then received GCSF (300 or 480 mcg according to weight SC daily for 3–5 days each week, starting 24 hours post-completion of EMA or CO | 1 (22) | |  |
|  | BEP | Cisplatin 20mg/m^2^ (30mg) IV and etoposide 70mg/m^2^ (100mg) IV on days 1–5, as well as bleomycin 10mg/m2 (15mg) intramuscular on days 1-3. The regimens were repeated every 21-28 days | 1 (66) | |  |
|  | FPV | Vincristine 1–2 mg through IV administration 12 h before 5-FU and cisplatin were administered on day 1; 5-FU 23–25 mg/(kg·d) through continuous IV administration, and 20 mg/(kg·d) cisplatin through an IV infusion on days 2–9 | 1 (67) | |  |
|  | Camrelizumab | Camrelizumab 200 mg IV every 2 weeks and apatinib 250 mg orally once per day | 1 (49) | |  |
|  | MAC | AC triple combination chemotherapy (MTX, 1 mg/kg IM on days1, 3, 5, and 7; folinic acid 0.1 mg/kg on days 2, 4, 6, and 8; Act-D 0.5 mg/day IV bolus for 5 days; cyclophosphamide 200 mg/day IV infusion for 5 days) | 1 (53) | |  |
| Other drugs referenced to treat GTN | | FAEV, VIP, TP/TE, DEMA/CO, EP, EMAEP + PC |  | |  |
| *Does not include cases of second-line therapy with Act-D/MTX following initial Act-D/MTX treatment.  5-FU, 5-fluorouracil; Act-D, actinomycin-D; BEP, bleomycin, etoposide, and cisplatin; d, day; DEMA/CO, dactinomycin, etoposide, methotrexate/cyclophosphamide, vincristine; EMA/CO, floxuridine, dactinomycin, and vincristine; etoposide, methotrexate, dactinomycin/cyclophosphamide, and vincristine; EMA-EP, etoposide, methotrexate, dactinomycin/etoposide, cisplatin; EP, etoposide, cisplatin; FAEV, floxuridine, dactinomycin, etoposide, and vincristine; GCSF, granulocyte-colony stimulating factor; GTN, gestational trophoblastic neoplasia; MAC, methotrexate, actinomycin D, and cyclophosphamide; ME-A, methotrexate, etoposide, dactinomycin; MTX, methotrexate; PC, paclitaxel-carboplatin; SC, subcutaneous; TP, paclitaxel and cisplatin; TP/TE, paclitaxel, cisplatin/paclitaxel, and etoposide; VIP, etoposide, ifosfamide, cisplatin. | | | | |  |

Table S5. Measures of treatment response reported from clinical trials.

Act-D, actinomycin-D; MTX, methotrexate.

|  |  | **No. of studies** | **Patients assessed for outcome** | **Successful treatment** | **Percentage, %** |
| --- | --- | --- | --- | --- | --- |
| All trials | All reported agents | 14 (38–50, 68) | 650 | 516 | 79.4 |
| First line | All reported agents | 9 | 617 | 475 | 77.0 |
| First line | MTX | 7 | 288 | 203 | 70.5 |
| First line | Act-D | 5 | 164 | 136 | 82.9 |

Table S6. Measures of any treatment response (unspecified) reported from observational studies.

| **Line of treatment** | **Drug** | **Risk** | **No. of studies reporting outcome** | **Patients at risk of outcome, n** | **Patients with outcome, n (%)** | **Percentage of patients with outcome in studies reporting outcome, mean (SD)** |
| --- | --- | --- | --- | --- | --- | --- |
| First | MTX | NR | 6 | 676 | 404 (59.76) | 404 (10.42) |
| Second | Act-D | High | 1 | 1 | 1 (100) | 100 (NA) |
| Second | Other | High | 4 | 101 | 32 (31.68) | 32 (39.57) |
| First | MTX | Low | 6 | 1,368 | 1,052 (76.9) | 1,052 (36.1) |
| NR | Other | High | 5 | 2,450 | 1,569 (64.04) | 1,569 (32.71) |
| First | Other | High | 9 | 603 | 354 (58.71) | 354 (34.38) |
| First | Other | NR | 10 | 630 | 367 (58.25) | 367 (36.89) |
| NR | Other | NR | 9 | 1,635 | 352 (21.53) | 352 (42.28) |
| First | Act-D | Low | 4 | 1,705 | 920 (53.96) | 920 (37.3) |
| Second | Other | Low | 3 | 61 | 50 (81.97) | 50 (15.28) |
| First | Other | Low | 6 | 399 | 295 (73.93) | 295 (25.23) |
| Second | Other | NR | 8 | 480 | 248 (51.67) | 248 (37.36) |
| First | Act-D | High | 2 | 2,001 | 774 (38.68) | 774 (36.32) |
| NR | Other | Low | 2 | 200 | 166 (83) | 166 (24.51) |
| First | Act-D | NR | 2 | 48 | 33 (68.75) | 33 (0) |

Act-D, actinomycin-D; MTX, methotrexate; NA, not applicable; NR, not reported; SD, standard deviation.

**Table S7.** Measures of treatment response (complete and partial) reported from observational studies.

| **Line of treatment** | **Drug** | **Risk** | **No. of studies reporting outcome** | **Patients at risk of outcome, n** | **Patients with outcome, n (%)** | **Percentage of patients with outcome in studies reporting outcome, mean (SD)** |
| --- | --- | --- | --- | --- | --- | --- |
| **Complete response** | | | | | | |
| NR | Other | High | 3 | 1,951 | 1,540 (78.93) | 63.89 (13.79) |
| First | Act-D | Low | 3 | 1,026 | 818 (79.73) | 77.71 (4.4) |
| First | MTX | Low | 3 | 978 | 705 (72.09) | 79.85 (17.77) |
| First | Other | Low | 2 | 93 | 78 (83.87) | 86.62 (14.65) |
| First | Act-D | High | 1 | 994 | 764 (76.86) | 76.86 (NA) |
| First | Other | NR | 3 | 204 | 182 (89.22) | 86.27 (10.55) |
| Second | Other | NR | 5 | 230 | 205 (89.13) | 72.1 (30.11) |
| First | Other | High | 3 | 233 | 217 (93.13) | 88.4 (14.27) |
| First | MTX | NR | 2 | 477 | 265 (55.56) | 62.95 (19.86) |
| First | Act-D | NR | 1 | 18 | 15 (83.33) | 83.33 (NA) |
| Second | Other | High | 1 | 15 | 12 (80) | 83.33 (23.57) |
| **Partial response** | | | | | | |
| First | Other | Low | 1 | 31 | 9 (29.03) | 29.03 (NA) |
| Second | Other | NR | 2 | 163 | 7 (4.29) | 9.37 (11.14) |
| First | Other | NR | 1 | 66 | 6 (9.09) | 9.09 (NA) |
| NR | Other | High | 1 | 35 | 3 (8.57) | 8.57 (NA) |

Act-D, actinomycin-D; MTX, methotrexate; NA, not applicable; NR, not reported; SD, standard deviation.

Table S8. Chemoresistance reported from observational studies.

| **Line of treatment** | **Drug** | **Risk** | **No. of studies reporting outcome** | **Patients at risk of outcome, n** | **Patients with outcome, n (%)** | **Percentage of patients with outcome in studies reporting outcome, mean (SD)** |
| --- | --- | --- | --- | --- | --- | --- |
| First | MTX | NR | 5 | 684 | 269 (39.33) | 33.86 (13.47) |
| First | Other | NR | 5 | 476 | 69 (14.5) | 13.26 (3.05) |
| NR | Other | NR | 2 | 1,073 | 147 (13.7) | 9.7 (5.86) |
| NR | Other | High | 3 | 475 | 229 (48.21) | 30.26 (18.28) |
| Second | Other | NR | 2 | 108 | 61 (56.48) | 52.56 (19.94) |
| First | Act-D | High | 1 | 994 | 48 (4.83) | 4.83 (NA) |
| First | Act-D | Low | 1 | 570 | 86 (15.09) | 16.09 (NA) |
| First | Other | High | 3 | 288 | 89 (30.9) | 30.03 (17.34) |
| First | Act-D | NR | 1 | 43 | 10 (23.26) | 22.36 (NA) |
| First | MTX | Low | 1 | 170 | 71 (41.76) | 41.76 (NA) |
| First | Other | Low | 2 | 276 | 87 (31.52) | 37.86 (17.17) |

Act-D, actinomycin-D; MTX, methotrexate; NA, not applicable; NR, not reported; SD, standard deviation.

Table S9. Mortality reported from observational studies.

| **Line of treatment** | **Drug** | **Risk** | **No. of studies reporting outcome** | **Patients at risk of outcome, n** | **Patients with outcome, n (%)** | **Percentage of patients with outcome in studies reporting outcome, mean (SD)** |
| --- | --- | --- | --- | --- | --- | --- |
| First | Other | NR | 2 | 106 | 9 (8.49) | 5 (7.07) |
| NR | Other | NR | 4 | 1204 | 39 (3.24) | 7.54 (7.94) |
| NR | Other | High | 1 | 431 | 3 (0.7) | 0.70 (NA) |
| First | Act-D | High | 2 | 1,007 | 10 (0.99) | 5.86 (9.37) |
| First | Act-D | Low | 1 | 570 | 0 (0) | 0 (NA) |
| First | Other | High | 2 | 107 | 11 (10.28) | 7.22 (6.74) |
| Second | Other | High | 1 | 59 | 2 (3.39) | 3.39 (NA) |
| First | MTX | Low | 1 | 20 | 2 (10) | 16.67 (23.57) |

Act-D, actinomycin-D; MTX, methotrexate; NA, not applicable; NR, not reported; SD, standard deviation.

Table S10. GTN recurrence reported from observational studies

| **Line of treatment** | **Drug** | **Risk** | **No. of studies reporting outcome** | **Patients at risk of outcome, n** | **Patients with outcome, n (%)** | **Percentage of patients with outcome in studies reporting outcome, mean (SD)** |
| --- | --- | --- | --- | --- | --- | --- |
| First | MTX | NR | 4 | 507 | 56 (11.05) | 18.69 (15.57) |
| NR | Other | High | 1 | 431 | 21 (4.87) | 4.87 (NA) |
| First | Other | NR | 4 | 92 | 16 (17.39) | 14.5 (11.62) |
| NR | Other | NR | 2 | 366 | 31 (8.47) | 16.34 (13.39) |
| First | Act-D | High | 2 | 1,007 | 28 (2.78) | 5.67 (7.59) |
| First | Act-D | Low | 1 | 570 | 15 (2.63) | 2.63 (NA) |
| Second | Other | NR | 2 | 173 | 49 (28.32) | 37.37 (23.3) |
| First | Other | High | 1 | 87 | 6 (6.9) | 7.5 (1.18) |
| First | MTX | Low | 2 | 190 | 7 (3.68) | 3.56 (3.57) |

Act-D, actinomycin-D; MTX, methotrexate; NA, not applicable; NR, not reported; SD, standard deviation.

Table S11. Summary of time to event outcomes associated with patient journey.

| **Interval** | **No. of studies** | **Mean** | **Median** | **Range** |
| --- | --- | --- | --- | --- |
| Pregnancy to diagnosis | 5*(76–80) | 39.3 days; SD 18.3 (mean of 3 studies reporting means/SD; 148 patients) | 324 days (mean of 4 studies reporting medians; 334 patients) | – |
| Pregnancy to treatment | 10** (80–89) | 413 days (mean of 6 studies reporting median; >508 patients, some studies reported NR) | 50 (median of 6 studies reporting medians; >508 patients, some studies reported NR) | 0–439 months |
| Diagnosis to treatment | 2***(90, 91) | 27.08 days (mean of 6 reported medians, 180 patients) | 26 days (median across 6 reported medians, 180 patients) | 0 – 91 days |
| *Six studies reported the outcome, and one presented as a categorical variable is not included in table; one study reported undetected lesions among 40 patients in China over a 23-year study period, increasing the overall time from pregnancy to diagnosis.  **Six studies reported statistics in continuous form.  ***Two studies consisting of six groups.  NR, not reported; SD, standard deviation. | | | | |

Table S12. Relevant GTN treatment guidelines included in the review

| **No.** | **Issuing body** | **Country** | **Year** | **Guideline focus** |
| --- | --- | --- | --- | --- |
| **1** | NCCN | USA | 2025 | GTN |
| **2** | Royal College of Obstetricians and Gynaecologists | UK | 2021 | GTD and GTN |
| **3** | Society of Obstetrician and Gynaecologists of Canada | Canada | 2021 | GTD and GTN |
| **4** | Guideline of the DGGG, OEGGG, and SGGG | DACH* | 2023 | GTN |
| **5** | Japan Society of Gynecologic Oncology | Japan | 2020 | Uterine body neoplasms |
| DGGG, Deutsche Gesellschaft für Gynäkologie und Geburtshilfe; GTD, gestational trophoblastic disease; GTN, gestational trophoblastic neoplasm;  NCCN, National Comprehensive Cancer Network; OEGGG, Österreichische Gesellschaft für Gynäkologie und Geburtshilfe; SGG, Schweizerische Gesellschaft für Gynäkologie und Geburtshilfe.  *Germany, Austria, and Switzerland. | | | | |

Table S13. Summary of recommendations from relevant GTN treatment guidelines

| **Category** | **Guidelines*** | **Type of GTN** | **Recommendation** |
| --- | --- | --- | --- |
| First-line treatment | 1, 2, 3, 4 | Low risk | Single-agent chemotherapy |
| First-line treatment | 1, 3, 4, 5 | High risk | EMA/CO |
| First-line treatment | 1, 2, 3, 4, 5 | PSTT, ETT | Surgery (1, 2, 5) and chemotherapy with a platinum-based regimen, such as EMA/EP, EP/EMA, or other regimens such as TP/TE, BEP, VIP, or ICE (1, 3, 4, 5) |
| Preferred drugs | 1, 3, 4 | Low risk | First line: MTX, Act-D |
| Preferred drugs | 1, 3, 4 | High risk | EMA/EP, EP/EMA, TP/TE, VIP, BEP, ICE, TIP |
| Additional drugs | 1 | High risk | PD-1/PD-L1 inhibitors (e.g., pembrolizumab [preferred], nivolumab [preferred], avelumab), nivolumab + ipilimumab, capecitabine-based regimens, gemcitabine ± cisplatin, high-dose chemotherapy with peripheral hematopoietic cell transplant (HCT), brain radiotherapy |
| Preferred drugs | 1 | PSTT, ETT | EMA/EP, EP/EMA, TP/TE (additionally VIP, BEP, CE) |
| Additional treatment options | 1, 2, 3, 4 | All risk | Recommendation for evaluation of immunotherapies as treatment, but the long-term effects of these treatments are unknown |
| Patient journey | 3 | High risk | Treatment at reference center |
| Monitoring | 1, 3, 4 | Not specified | hCG monitoring every 1-2 weeks during treatment; 1 time per month for 1-2 years following treatment |
| Monitoring | 1, 3, 4 | Not specified | Consolidation therapy 2-4 cycles |
| Patient journey | 2 | Not specified | Increased risk of early menopause for women with multiagent chemotherapy |
| Patient journey | 2 | Not specified | Pregnancies achieved in 80% of women following chemotherapy |
| Act-D, dactinomycin; BEP, bleomycin, etoposide, cisplatin; EMA/CO, etoposide, methotrexate, dactinomycin/cyclophosphamide, vincristine; EMA/EP, etoposide, methotrexate, dactinomycin/etoposide, cisplatin; EP/EMA, etoposide, cisplatin/etoposide, methotrexate, actinomycin-D; ETT, epithelioid trophoblastic tumors; hCG, human chorionic gonadotropin; ICE, ifosfamide, carboplatin, etoposide; MTX, methotrexate; PSTT, placental-site trophoblastic tumors; TIP, paclitaxel, ifosfamide, cisplatin; TP/TE, paclitaxel, cisplatin/paclitaxel, etoposide; VIP, etoposide, ifosfamide, cisplatin.  * Refer to numbering in Table S12. | | | |
|  |  |  |  |
|  |  |  |  |

Table S14. Distribution of adverse events reported from clinical trials.

| **Treatment regimen** | **Patients, n** | **Alopecia** | | **Fatigue** | | **Nausea** | | **Gastrointestinal (excluding nausea related)** | | **Hematologic** | |
| --- | --- | --- | --- | --- | --- | --- | --- | --- | --- | --- | --- |
|  |  | n | % | n | % | n | % | n | % | n | % |
| MTX | 208 | 12 | 5.8 | 57 | 27.4 | 32 | 15.4 | 75 | 36.1 | 15 | 7.2 |
| Act-D | 199 | 14 | 7.0 | 58 | 29.1 | 56 | 28.1 | 79 | 39.7 | 16 | 8.0 |
| MTX + Act-D | 50 | 0 | 0 | NR | 5.95 | 8 | 16 | 6 | 12 | 1 | 2 |
| EMA/CO | 43 | NR | NR | NR | NR | NR | NR | NR | NR | 14 | 32.6 |
| FAEV | 46 | NR | NR | NR | NR | NR | NR | NR | NR | 28 | 60.1 |

Act-D, dactinomycin; EMA/CO, etoposide, methotrexate, dactinomycin/cyclophosphamide, vincristine; FAEV, floxuridine, dactinomycin, etoposide, and vincristine; MTX, methotrexate; NR, not reported.

Figures

Figure S1. Summary of continuous age reported from 53 studies included in the SLR.

IQR, interquartile range; SLR, systematic literature review.

Figure S2. Summary of baseline FIGO stage from 47 reporting studies, grouped and single stage.

FIGO, International Federation of Gynecology and Obstetrics.

Figure S3. Distribution of quality assessment ratings by study type.
